# Simulation and optimization of nutrient uptake and biomass formation using a multi-parameter Monod-type model of tobacco BY-2 cell suspension cultures in a stirred-tank bioreactor

**DOI:** 10.3389/fpls.2023.1183254

**Published:** 2023-10-31

**Authors:** Henrik Nausch, Marco Baldan, Katrin Teichert, Jannik Lutz, Carsten Claussen, Michael Bortz, Johannes Felix Buyel

**Affiliations:** ^1^ Department Bioprocess Engineering, Fraunhofer Institute for Molecular Biology and Applied Ecology IME, Aachen, Germany; ^2^ Division Optimization, Fraunhofer Institute for Industrial Mathematics ITWM, Kaiserslautern, Germany; ^3^ Fraunhofer Institute for Translational Medicine and Pharmacology ITMP, Hamburg, Germany; ^4^ Institute for Molecular Biotechnology, RWTH Aachen University, Aachen, Germany; ^5^ Institute of Bioprocess Science and Engineering (IBSE), University of Natural Resources and Life Sciences, Vienna (BOKU), Vienna, Austria

**Keywords:** biopharmaceuticals, cultivation medium, mechanistic model, multi-criteria optimization, upstream production

## Abstract

**Introduction:**

Tobacco (*Nicotiana tabacum*) cv Bright Yellow-2 (BY-2) cell suspension cultures enable the rapid production of complex protein-based biopharmaceuticals but currently achieve low volumetric productivity due to slow biomass formation. The biomass yield can be improved with tailored media, which can be designed either by laborious trial-and-error experiments or systematic, rational design using mechanistic models, linking nutrient consumption and biomass formation.

**Methods:**

Here we developed an iterative experiment-modeling-optimization workflow to gradually refine such a model and its predictions, based on collected data concerning BY-2 cell macronutrient consumption (sucrose, ammonium, nitrate and phosphate) and biomass formation.

**Results and discussion:**

The biomass formation was well predicted by an unstructured segregated mechanistic Monod-type model as long as the nutrient concentrations did not approach zero (we omitted phosphate, which was completely depleted). Multi-criteria optimization for sucrose and biomass formation indicated the best tradeoff (in a Paretian sense) between maximum biomass yield and minimum process time by reducing the initial sucrose concentration, whereas the inoculation biomass could be increased to maximize the biomass yield or minimize the process time, which we confirmed in calibration experiments. The model became inaccurate at biomass densities > 8 g L^-1^ dry mass when sucrose was almost depleted. We compensated for this limitation by including glucose and fructose as sucrose hydrolysis products in the model. The remaining offset between the simulation and experimental data might be resolved by including intracellular pools of sucrose, ammonium, nitrate and phosphate. Overall, we demonstrated that iterative models can be used to systematically optimize conditions for bioreactor-based processes.

## Introduction

Plant cell cultures (PCCs) can be used to produce complex protein-based biopharmaceuticals such as growth factors, cytokines or antibodies, especially proteins that are unsuitable for expression in microbial or mammalian cells (Xu and Zhang, 2014; Santos et al., 2016). PCCs from carrot (*Daucus carrota*), rice (*Oryza sativa*) and tobacco (*Nicotiana tabacum*), in particular tobacco cell lines NT-1 and BY-2, are often used for this purpose (Xu et al., 2011; Xu and Zhang, 2014; Santos et al., 2016; Moon et al., 2020). Tobacco cells have short doubling times of 25–30 h compared to 50–500 h for other PCCs and can be grown to high cell densities of up to 300–600 g L^-1^ fresh mass (FM) in large-scale stirred-tank reactors (STRs) with working volumes of up to 100,000 L (Fischer et al., 1999). However, PCCs generally have a low productivity of up to 1 g L^-1^ of protein, compared to 5–20 g L^-1^ for microbial and mammalian cells (Schillberg and Spiegel, 2022).

One option to increase the productivity of PCCs is to optimize the growth medium (Terashima et al., 2001; Holland et al., 2010; Holland, 2013; Vasilev et al., 2013; Häkkinen et al., 2018; Sadoch et al., 2020). PCCs are typically cultivated in chemically defined media containing sucrose (C_12_H_22_O_11_) as a carbon source, ammonium nitrate (NH_4_NO_3_) and/or potassium nitrate (KNO_3_) as nitrogen sources, and potassium dihydrogen phosphate (KH_2_PO_4_) as a phosphate source. The feeding strategy also affects the volumetric productivity. For example, varying nitrogen sources in trial-and-error screening experiments led to recipes such as Murashige and Skoog (MS) medium (Murashige and Skoog, 1962), Gamborg B5 (Gamborg et al., 1968), Chu N6 (Chu et al., 1975), Schenk and Hildebrandt (SH) medium (Schenk and Hildebrandt, 1972), and amino acid (AA) medium (Thompson et al., 1986). More recently, carbon, nitrogen and phosphate sources have been optimized more systematically using a Design of Experiments (DoE) approach, confirming the potential to increase biomass formation and volumetric productivity of protein-based biopharmaceuticals. These studies also demonstrated that mineral salts, vitamins and plant growth regulators, including substances referred to as phytohormones, are required for the growth of PCCs, but have only a minor impact on biomass formation and biopharmaceutical production (Holland, 2013; Vasilev et al., 2013). Importantly, the experiments were conducted in shake flasks and the transferability of such conditions to STRs has yet to be demonstrated.

Model-based optimization is another systematic option to improve media composition (Schinn et al., 2021; Yeo et al., 2022). Model-based DoE methods attempt to find new sets of experiments that improve the model based on prior knowledge about the system and a small number of initial tests (Seufert et al., 2021). In general, there are three modeling strategies: data-driven (black box), mechanistic (white box), and hybrid (gray box). The mechanistic modeling are based on physical, chemical and biological principles that describe the interdependency of parameters such as nutrient uptake, biomass formation and target protein production, thus enabling robust bioprocess engineering (Tsopanoglou and Del Jiménez Val, 2021). Additionally, mechanistic models can be described as structured or unstructured. Whereas unstructured models consider nutrient (substrate) uptake from the medium, biomass formation and protein production, structured models also include the intracellular metabolism of nutrients. Compared to structured models, unstructured models require a limited number of equations and parameters, making them less prone to overfitting when presented with limited input data (Hawkins, 2004). Both unstructured and structured models can be either unsegregated, which assumes the cell population is homogeneous, or segregated, which differentiates cells into viable and dead cells or differentiates by nutrient uptake, biomass formation, and protein production.

A mechanistic, kinetic modeling approach is preferred for PCCs, where all metabolic effects are lumped into unstructured kinetic functions for simplification (Resat et al., 2009), especially for model-based DoE where the number of experiments is small and hence only few model parameters can be fitted. In particular, Monod-type models are preferred because they simulate nutrient uptake, biomass formation and protein production while differentiating between viable and dead cells (Prakash and Srivastava, 2006; Prakash and Srivastava, 2008; Jiménez-Hornero et al., 2009a; Jiménez-Hornero et al., 2009b; Puad et al., 2017; Villegas et al., 2017; Puad and Abdullah, 2018; Jacob et al., 2020).

**Figure 1:**
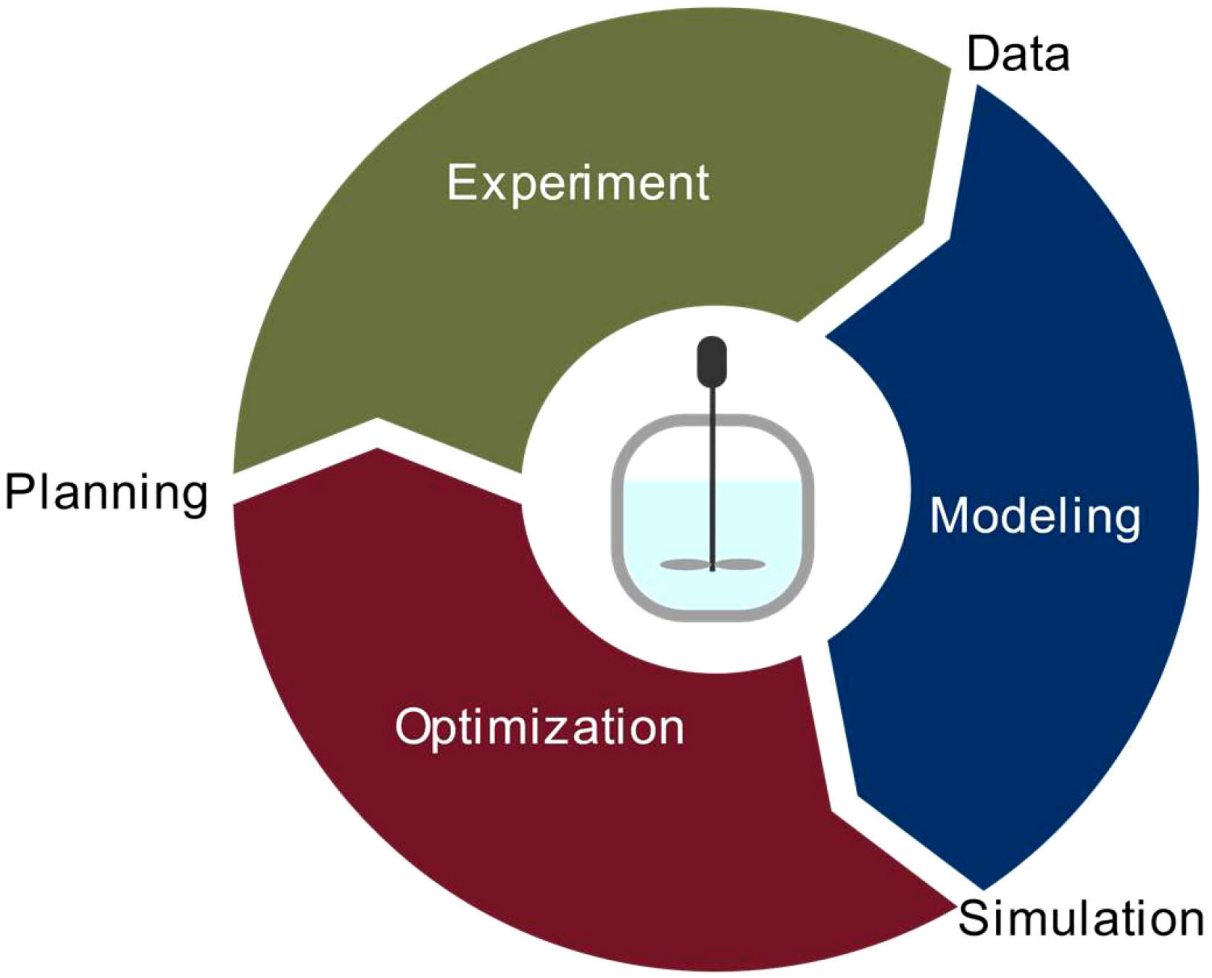
Iterative experiment-model-optimization workflow. In the first iteration, initial experiments were carried out to collect starting data (e.g., for nutrient consumption, cell growth and biomass formation), a model was set up in order to identify relevant parameters (e.g., nutrients), and the model, once embedded in an optimization frame, was used to predict optimal process conditions (e.g., initial nutrient concentrations). In the second iteration, these optimized process conditions were experimentally verified and used to update the model calibration and/or process and for model improvement.

Model-based optimization requires an iterative workflow for process characterization and model setup followed by process and model improvement in three steps: experiment, modeling, and optimization (**Figure 1**). A similar workflow has been applied in chemical engineering (Höller et al., 2019; Asprion et al., 2022). In the first iteration, initial experiments are carried out to collect starting data, e.g., a few experiments to characterize nutrient consumption, cell growth and biomass formation. Then a model is set up to identify relevant parameters (e.g., concentration of specific nutrients), and the model is used to predict improved/optimal process conditions, e.g., altered initial nutrient concentrations (**Table S1**). In the second iteration, these optimized process conditions are experimentally verified and used to update the model calibration and/or process for further model improvement (**Table S1**). Both, the process and model, can then be refined in subsequent iterations until the desired model fidelity or process improvement is achieved.

Here we applied an unstructured segregated Monod-type model to describe the nutrient uptake and biomass formation of tobacco BY-2 cells as model PCC in 2-L and 5-L STRs as part of a semi-continuous fermentation process (Patent WO2015165583A1). We used the model to maximize the biomass yield and minimize the process time by medium optimization. The purpose of the study was to demonstrate that relevant bioprocess optimization is possible using a very small number of experiments in combination with corresponding models.

## Materials and methods

### Cultivation of tobacco cells

Tobacco (*N. tabacum* BY-2) cells were grown in either 100-mL or 1000-mL Erlenmeyer shake flasks containing 20 mL or 200 mL of modified MS medium as previously described (Murashige and Skoog, 1962; Rademacher et al., 2019). Flasks were placed on a Climo-Shaker ISF1-X orbital shaker (Kuhner Shaker, Herzogenrath, Germany) at 26°C, shaking at 160 rpm with a displacement of 25 mm. The BY-2 cells were transferred to fresh MS medium every 7 days using an inoculation cell density of 20 g L^-1^ FM.

The 7-day-old cells were used to inoculate MS medium in 2-L or 5-L STRs (Getinge Deutschland, Rastatt, Germany) (**Tables S2**, **S3**). The double-walled glass STRs were equipped with dissolved oxygen (dO_2_), capacitance, and pH probes, and contained a porous sparger for aeration. Two axial marine and two radial flat-blade impellers were used for mixing. The bottom marine impeller was installed at a height corresponding to the mid-level of the minimum culture volume, whereas the second marine impeller was placed at the corresponding height of the maximum culture volume (**Figure S1**). The bottom flat-blade impeller was installed equidistant in between the two marine impellers. The second flat-blade impeller was positioned at the same distance above the top marine impeller. The dO_2_ level was kept constant by controlling the stirrer speed. The pH were monitored but not controlled. Online data were recorded using the BioExpert process information management system (Getinge Deutschland). The FM data were used to calibrate the capacitance probe that controlled the feeding rate. The concentration of sucrose, glucose and fructose in the medium was adjusted for each fermentation run as described below (**Table S3**). The feed phase was initiated at a FM of 100 g L^-1^ and this FM concentration was maintained by adding fresh medium. In addition, interspersed draining of cultivation broth was used every 22–26 h to restore the starting volume. The feed medium was of the same composition as the batch medium but contained 59 mM (or 20 g L^-1^) instead of 88 mM (or 30 g L^-1^) sucrose. The feed rate was dynamically adjusted in dependence of the growth rate that was monitored via a capacitance probe. Accordingly, the drain volumes differed over time. Process samples were taken for subsequent analysis every 22–26 h (simultaneously with draining during the semi-continuous phase). A 5-L bioreactor was used in the initial experiments (#1-3) for model setup, whereas 2-L reactors were used for validation experiments (#4-9). The feeding and control strategy as well as the impeller configuration were the same in both cases.

### Cell biomass and macronutrient analysis

To determine the BY-2 cell concentration, each 100-µL sample was diluted 1:10 by mixing with 1 mL 0.9% (m v^-1^) sodium chloride containing 0.025% (m v^-1^) Evans blue, and the cells were counted in a Fuchs-Rosenthal counting chamber. To determine the FM, DM and macronutrient levels, 5.0-mL samples were applied to cellulose filter paper in a Büchner funnel, and the medium removed by vacuum filtration at 0.08 MPa for 5 s. For the FM, the BY-2 pellet was transferred to a weighing dish and the FM was determined using a fine balance. For the dry mass (DM), the weighing dish with the BY-2 pellet was dried at 60°C for 3 days and weighed again. For the macronutrients, the flow-through, obtained from the vacuum filtration, was collected in a 15-mL Falcon tube and stored at –20°C before analysis using commercial assay kits for sucrose (cat. MAK013; Merck, Darmstadt, Germany), glucose (cat. ABIN5067615; antibodies-online, Aachen, Germany), fructose (cat. K619-100; BioVision, Ilmenau, Germany), ammonium (cat. MAK310; Merck), nitrate (cat. Cay780001; Biomol, Hamburg, Germany), and phosphate (cat. KA0815; Abnova Germany, Heidelberg, Germany).

### Model fitting and multi-criteria optimization

Calculations were performed using the dynamic programming language Julia^1^. The optimization solver was Ipopt (Wächter and Biegler, 2006). We used the default values of the termination criteria, i.e., the (scaled) non-linear problem error (<10^-8^) and the (absolute) criteria according to “dual_inf_tol” (1), “constr_viol_tol” (10^-4^), and “compl_inf_tol” (10^-4^) ^2^. The performance of different models was compared by K-fold cross-validation (Stone, 1974). Confidence intervals (CIs) were calculated based on bootstrapping (Dogan, 2007). Inequality constraints were used in the parameter identification problem to ensure that values of inhibition constants were at least equal to or more extreme than the corresponding saturation constants.

## Results and discussion

### Phosphate is rapidly depleted from the PCC medium whereas sucrose, ammonium and nitrate are not completely consumed

**Figure 2:**
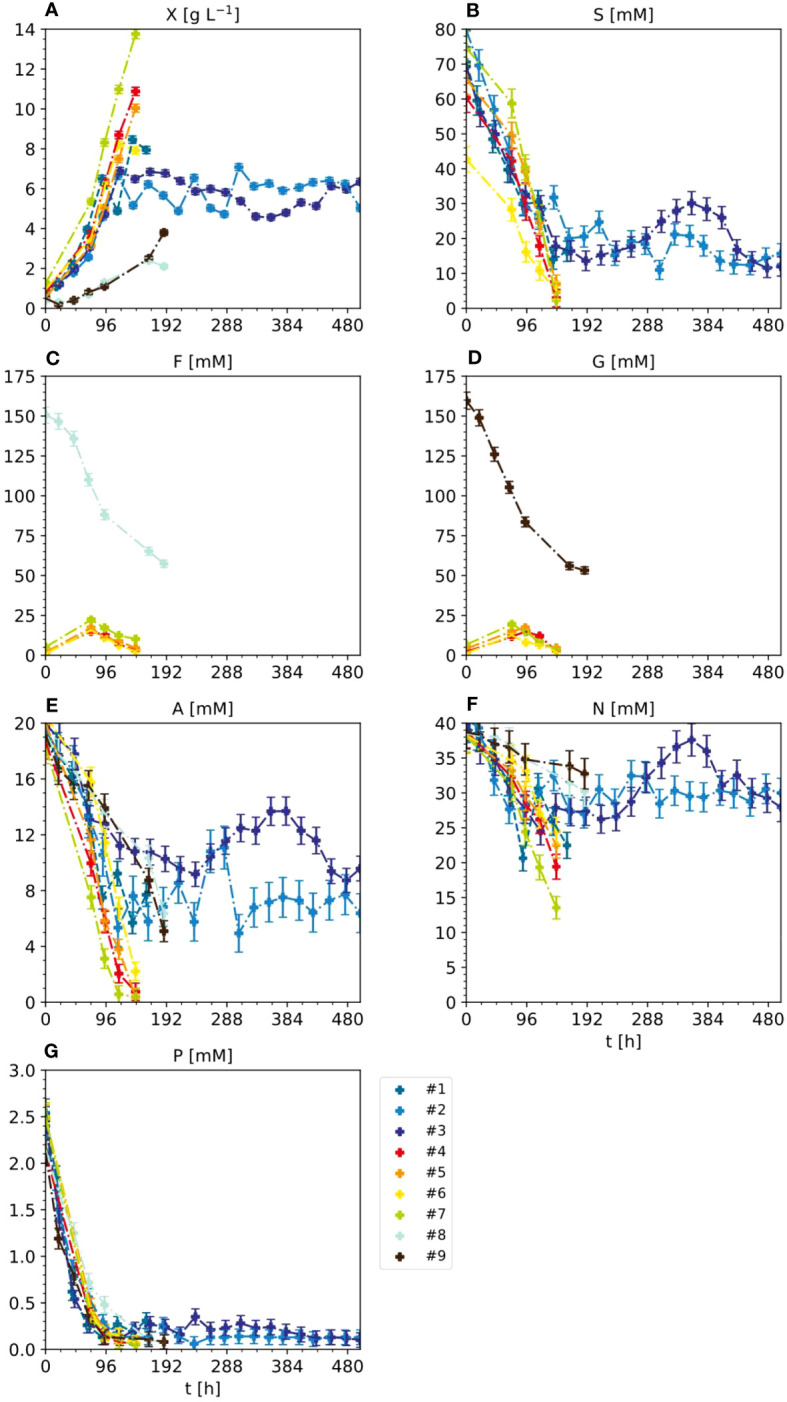
Nutrient consumption and cell growth/biomass formation under standard, optimal and non-optimal cultivation conditions. Experiments 1–3: standard cultivation conditions used for model setup (iteration 1). Experiments 4 and 5: optimal cultivation conditions according to the model-based optimization for model validation (iteration 2). Experiments 6 and 7: non-optimal cultivation conditions used for model validation (iteration 2). Experiments 8 and 9: non-optimal cultivation conditions but different carbon sources (either fructose or glucose instead of sucrose) used for model improvement (iteration 2). Measurement uncertainty made of a constant and proportional component (**Table S4**). Numbers – individual experiments- **(A)** Dry mass X. **(B)** Sucrose S. **(C)** Fructose **(F, D)** Glucose **(G, E)** Ammonium **(A, F)** Nitrate N. **(G)** phosphate P.

In the standard setting for the BY-2 semi-continuous fermentation (experiments #1, #2 and #3) (**Figure 2** and **S2**, **Table S3**), BY-2 cells were cultivated in MS medium in 5-L STRs, starting with a cell density of 20 ± 5 g L^-1^ FM (corresponding to 0.75 ± 0.22 g L^-1^ DM) at the beginning of the batch phase, and were kept at 100 ± 20 g L^-1^ FM during the semi-continuous phase. Under these conditions, the initial sucrose concentration of 88 mM decreased to 25–30 mM at the end of the batch phase and remained at 15–30 mM during the semi-continuous phase, which correlated with the slight variation in the FM/DM ratios (**Figure S2**). In line with the declining concentration of sucrose, the concentration of its hydrolysis product glucose increased from 0 mM at t_0_ to 15 mM at the end of the batch phase, and remained at 12–18 mM in the semi-continuous phase. This is consistent with the fact that sucrose is not only taken up by plant cells via sucrose transporters, but is also hydrolyzed into glucose and fructose externally by invertases bound to the cell wall, and these products are then taken up by hexose transporters (Lemoine et al., 2013).

Like sucrose, ammonium levels decreased from 21 to 10–12 mM at the end of the batch phase and stayed at 5–10 mM during the semi-continuous phase, correlating with the FM/DM. The decrease in ammonium may reflect its uptake by ammonium transporters and the intracellular detoxification of ammonium via the glutamine synthase/glutamine oxoglutarate aminotransferase (GS/GOGAT) cycle that produces glutamate (Bittsánszky et al., 2015). To ensure ammonium levels remain sub-toxic, other PCC media including Gamborg B5, Chu N6, SH medium and AA medium contain 2, 7, 3 and 0 mM ammonium, respectively, compared to the 21 mM present in MS medium. However, Ullisch (2012) observed that increasing the ammonium concentration in MS medium from 21 to 41 mM caused NT-1 to accumulate 20% more biomass, possibly due to the formation of more glutamate (Ullisch, 2012). Even though not all the ammonium is consumed, it may therefore be a suitable target for medium optimization when aiming to accelerate biomass formation.

The decrease in nitrate levels was much less pronounced than the other components, falling from an initial 39 mM to 25–30 mM at the end of the batch phase, which was maintained during the semi-continuous phase. This small change in nitrate levels may reflect the conversion of nitrate into ammonium by nitrate reductase and nitrite reductase, which are subject to feedback inhibition to ensure ammonium stays at sub-toxic levels (Behrend and Mateles, 1975; Behrend and Mateles, 1976). Notably, increasing nitrate levels from 39 to 139 mM reduced growth by 30%, and when nitrate was the sole nitrogen source the plant cells did not grow at all (Holland et al., 2010; Ullisch et al., 2012).

In contrast to sucrose, ammonium and nitrate, the initial 2.7 mM phosphate present in the MS medium was almost completely consumed within 3 days of batch fermentation and remained at 0.1–0.3 mM during the semi-continuous phase. This agrees with previous studies reporting that phosphate is a growth-limiting factor during the fermentation of BY-2 cells, even if the phosphate concentration is increased by 400% (Holland, 2013; Vasilev et al., 2013).

Therefore, the initial set of experiments yielded plausible data that we used for an initial model calibration.

### The model for the standard cultivation conditions in STRs suggests that sucrose levels can be reduced without affecting biomass formation and volumetric biomass yield

For the standard BY-2 semi-continuous fermentation in STRs (experiments #1, #2 and #3) (**Figure 2** and **S2**), we applied a Monod-type unstructured segregated model to describe the extracellular dynamics of cell growth/biomass formation (DM increase over time) and nutrient uptake. As proposed by Jacobs et al. (2020), we modeled two physiological states of cell DM: viable (active) (*X_a_*) and dead (*X_d_*). This yields the total DM concentration *X* [g L^-1^] (Eq. 1).


(1) $$X=X_{a}+X_{d}$$

The model assumes an irreversible progression in which active cells (active DM) are converted into dead cells (dead DM) (Jacob et al., 2020) (Eq. 2).


(2) $$V\frac{dX_{a}}{dt}+X_{a}\frac{dV}{dt}=\mu X_{a}V-k_{d}X_{a}V$$

where *μ* [h^-1^] is the specific growth rate,*k_d_* [h^-1^] is the death rate constant of active DM, and *V* [L] is the volume of the cultivation medium. Based on the volumetric change of the cultivation medium, we calculated the feed rate *F_t_* [L h^-1^] for each time point *t* (Jiménez-Hornero et al., 2009a) (Eq. 3).


(3) $$\frac{dV}{dt}=F_{t}$$

The dependency of the specific growth rate (biomass formation) of active DM (*μ*) on sucrose (*S*), ammonium (*A*) and nitrate (*N*) concentrations can be expressed as a Monod kinetic (Eq. 4).


(4) $$\mu =\mu_{m}\;\left(\frac{S}{S+K_{S}}\frac{K_{IS}}{S+K_{IS}}\right)\left(\frac{A}{A+K_{A}}\frac{K_{IA}}{A+K_{IA}}\right)\left(\frac{N}{N+K_{N}}\frac{K_{IN}}{N+K_{IN}}\right)$$

where *K_r_* is the saturation constant and *K_Ir_* the inhibition constant of corresponding nutrient *r*, i.e. *S, A* and *N* in [g L^-1^], and μ_m_ is the maximum specific growth rate [h^-1^] (Prakash and Srivastava, 2006; Prakash and Srivastava, 2008). For each nutrient, we used inequality constraints to force the inhibition constant to be equal to or greater than the saturation constant during parameter identification to enforce actual plateaus, i.e., saturation.

The phosphate concentration was modeled but its effect was not included in the specific growth rate because it dropped below the limit of quantification after 3 days of batch cultivation. Accordingly, adding a term for the phosphate concentration in the specific growth rate (Eq. 4) would have zeroed out the DM growth after 3 days, which did not agree with the observed DM increase. This limitation of the current model might be circumvented in the future by including intracellular phosphate levels in a structured model but the determination of such pools was beyond the scope of this study.

The sucrose, ammonium, nitrate and phosphate concentrations during BY-2 cultivation were modeled in Equations 5–8.


(5) $$V\frac{dS}{dt}+S\frac{dV}{dt}=F_{t}S_{f}-\mu_{S}\left(\frac{S}{S+K_{S}}\frac{K_{IS}}{S+K_{IS}}\right)X_{a}V$$

(6) $$V\frac{dN}{dt}+N\frac{dV}{dt}=F_{t}N_{f}-\mu_{N}\left(\frac{N}{N+K_{N}}\frac{K_{IN}}{N+K_{IN}}\right)X_{a}V$$

(7) $$V\frac{dA}{dt}+A\frac{dV}{dt}=F_{t}A_{f}-\mu_{A}\left(\frac{A}{A+K_{A}}\frac{K_{IA}}{A+K_{IA}}\right)X_{a}V$$

(8) $$V\frac{dP}{dt}+P\frac{dV}{dt}=F_{t}P_{f}-\mu_{P}P\;V$$

where *S_f_, N_f,_ A_f_* and *P_f_* are the feed medium concentrations of sucrose, nitrate, ammonium and phosphate respectively, and *μ_S,_ μ_N,_ μ_A_* and *μ_P_* denote the corresponding sucrose, nitrate, ammonium and phosphate consumption rates [h^-1^]. As for equation 4, the influence of the active DM (X_a_) on the phosphate uptake in (Eq. 8) was neglected because i) the phosphate concentration dropped close to zero within ~90 h (**Figure 2**) and the “0” values would compromise numeric stability of the resulting models and ii) the necessary additional parameter would increase the likelihood of overfitting given the small calibration data set (n=3). As above, potential intracellular phosphate pools might be accounted for in future studies to refine our model, the measurement of such pools was beyond the scope of this study where we intended to demonstrate rapid optimization potentials for bioprocesses operation with a minimal number of experiments.

Parameter identification focused on calibrating the 12 parameters (**Table 1**) of the model (1)-(8), which is described hereafter as the “initial model”.

**Table 1:** Parameter ranges and identified optimal parameter values.

Parameter	Description	Unit	Min	Max	Optimal	90% CI
$k_{d}$	Death rate constant	h^-1^	0	0.1	6.62 × 10^-4^	5.8 × 10^-5^
$\mu_{m}$	Maximum specific growth rate	h^-1^	0	10	9.47	0.06
$K_{S}$	Sucrose saturation constant	g L^-1^	0	100	17.64	3.5
$K_{IS}$	Sucrose inhibition constant	g L^-1^	0	100	17.64	3.4
$K_{A}$	Ammonium saturation constant	g L^-1^	0	1	0.796	0.040
$K_{IA}$	Ammonium inhibition constant	g L^-1^	0	1	0.797	0.024
$K_{N}$	Nitrate saturation constant	g L^-1^	0	5	0.133	0.057
$K_{IN}$	Nitrate inhibition constant	g L^-1^	0	5	0.135	0.097
$\mu_{s}$	Sucrose consumption rate	h^-1^	0	1	2.235 × 10^-1^	4.65 × 10^-2^
$\mu_{A}$	Ammonium consumption rate	h^-1^	0	1	4.863 × 10^-3^	6.34 × 10^-4^
$\mu_{N}$	Nitrate consumption rate	h^-1^	0	1	3.013 × 10^-2^	2.49 × 10^-3^
..	Phosphate consumption rate	h^-1^	0	1	2.883 × 10^-2^	2.36 10^-3^

Model parameters can be identified by minimizing an objective function dependent on a norm for the error made in measuring the process outputs (Jiménez-Hornero et al., 2009b). Here, we used the weighted least-squares function as an objective function, which for normally distributed errors constitutes a maximum likelihood estimator (Eq. 9 and 10).

$\theta^{*}=\underset{\theta }{\min}Q\left(\theta ;\tilde{x},\tilde{t},\tilde{y}\right)=\sum_{h=1}^{H}\sum_{t=1}^{T\left(h\right)}q_{h,t}\left(\theta ;{\tilde{x}}_{h},{\tilde{t}}_{h,t},{\tilde{y}}_{h,t}\right)$ (9)


(10) $$q_{h,t}\left(\theta ;{\tilde{x}}_{h},{\tilde{t}}_{h,t},{\tilde{y}}_{h,t}\right)=\sum_{r=1}^{R\left(h,t\right)}w_{r}{\left[1-\frac{g_{r}\left(\theta ;{\tilde{x}}_{h},{\tilde{t}}_{h,t}\right)}{{\tilde{y}}_{h,t,r}}\right]}^{2}$$

where index *t = 1, …, T* denotes the time points at which the individual model factors *r = 1, …, R* (i.e. ammonium, …, sucrose as well as the DM) were measured for each experiment *h = 1, …, H* which created $\tilde{t},\tilde{y}$ pairs ( $\tilde{t}$ is the time of measurement and $\tilde{y}$ denotes the measured value). The model solution was synthetically described by *y = g(θ; t)* and had *R* responses (in this case represented by nutrients and DM). The term *w* ∈ ℝ^R^ is a vector of weights for these responses to balance their priorities. Here, we used equal values, i.e., the unit vector, giving equal weights for each response. *θ* represents the model parameters (**Table 1** for the initial model).

The CI for parameter estimates was processed using a residual-based non-parametric bootstrap resampling approach (Dogan, 2007). The error matrix Σ was obtained by the measured data ${\tilde{y}}_{\text{h},\text{t},\text{r}}$ . Specifically, the entries of the matrix were the absolute and proportional measurement uncertainties u_r_^0^ and u_r_^%^, respectively, that were determined for each model factor r (**Table S4**).


(11) $$\sum =\left[\begin{array}{cccc} {\text{u}}_{1}^{0}+{\text{u}}_{1}^{\%}{\tilde{y}}_{1,1,1} & \ldots & {\text{u}}_{1}^{0}+{\text{u}}_{1}^{\%}{\tilde{y}}_{t,h,1} & \ldots \\ {\text{u}}_{2}^{0}+{\text{u}}_{2}^{\%}{\tilde{y}}_{1,1,2} & \ldots & {\text{u}}_{2}^{0}+{\text{u}}_{2}^{\%}{\tilde{y}}_{h,t,2,} & \ldots \\ \ldots & \ldots & \ldots & \ldots \\ {\text{u}}_{\text{R}}^{0}+{\text{u}}_{\text{R}}^{\%}{\tilde{y}}_{1,1,R} & \ldots & {\text{u}}_{\text{R}}^{0}+{\text{u}}_{\text{R}}^{\%}{\tilde{y}}_{h,t,R} & \ldots \end{array}\right]$$

This approach for error estimation is preferred over the more widely used model-data mismatch because the measurement error is assumed known (**Table S4**) (Joshi et al., 2006). The error values for model factors were resampled with replacement to create a large pool of error matrix sets (~1,000). Subsequently, each of the resampled error matrices was added back to the original modeled data (i.e., model prediction) to create a pool of synthetic data sets. Each of the generated data sets was independently processed through least-squares estimation (Eq. 9 and 10) to identify a set of model parameter values that formed the basis for CI calculation as described elsewhere (Dekking et al., 2005).

The quality measure of the model prediction for the r*-th* output (i.e., model factor) was quantified by the mean average error MAE (Borchani et al., 2015), which measures the average error between model prediction g and data $\tilde{\mathrm{y.}}$


(12) $$MAE_{r}^{h}\left(\theta^{*}\right)=\frac{1}{T\left(h\right)}\sum_{t=1}^{T\left(h\right)}\left|{\tilde{y}}_{h,t,r}-g_{r}\left(\theta^{*};{\tilde{x}}_{h},{\tilde{t}}_{h,t}\right)\right|$$

Moreover, we introduced a second quality measure (nMAE) corresponding to the MAE normalized with respect to the available data in order to normalize the data from experiments #1 to #7 (i.e., $\bar{H}\;$ = 7).


(13) $$nMAE_{r}^{h}\left(\theta^{*}\right)=\frac{MAE_{r}^{h}\left(\theta^{*}\right)}{\frac{1}{\sum_{k=1}^{\bar{H}}T\left(k\right)\;}\sum_{h=1}^{\bar{H}}\sum_{t=1}^{T\left(j\right)}{\tilde{y}}_{h,t,r}}=\frac{MAE_{r}^{h}\left(\theta^{*}\right)}{{\bar{y}}_{r}}\;$$

The indices *h* and *k* indicate subsets of the dataset when more than one dataset D={1,2,.}⊆{1,…,9} was considered at the same time:


(14) $$MAE_{r}^{D}\left(\theta^{*}\right)=\frac{1}{\sum_{k\in D}T\left(k\right)}\sum_{h\in D}\sum_{t=1}^{T(h)}\left|{\tilde{y}}_{h,t,r}-g_{r}\left(\theta^{*};{\tilde{x}}_{h},{\tilde{t}}_{h,t}\right)\right|$$

The initial mass concentration of carbohydrates was 30 g L^-1^ in all experiments. In experiments #1-7 carbohydrates were provided as 88 mM sucrose whereas it was 151 mM fructose in #8 and 151 mM glucose in #9. Accordingly, experiments #1 - #7 did not contain fructose or glucose at the start of the fermentation. Instead, the monosaccharides formed in these experiments due to enzymatic cleavage of sucrose during fermentation. Because glucose was rapidly taken up by the BY-2 cells, the glucose concentrations in the culture medium of #1-7 was only in the 15–21 mM range. This was approximately 10-fold lower than the 151 mM fructose and glucose present at the start of experiments #8 and #9 respectively. Therefore, experiments #8 and #9 were not considered during this analysis.

**Figure 3:**
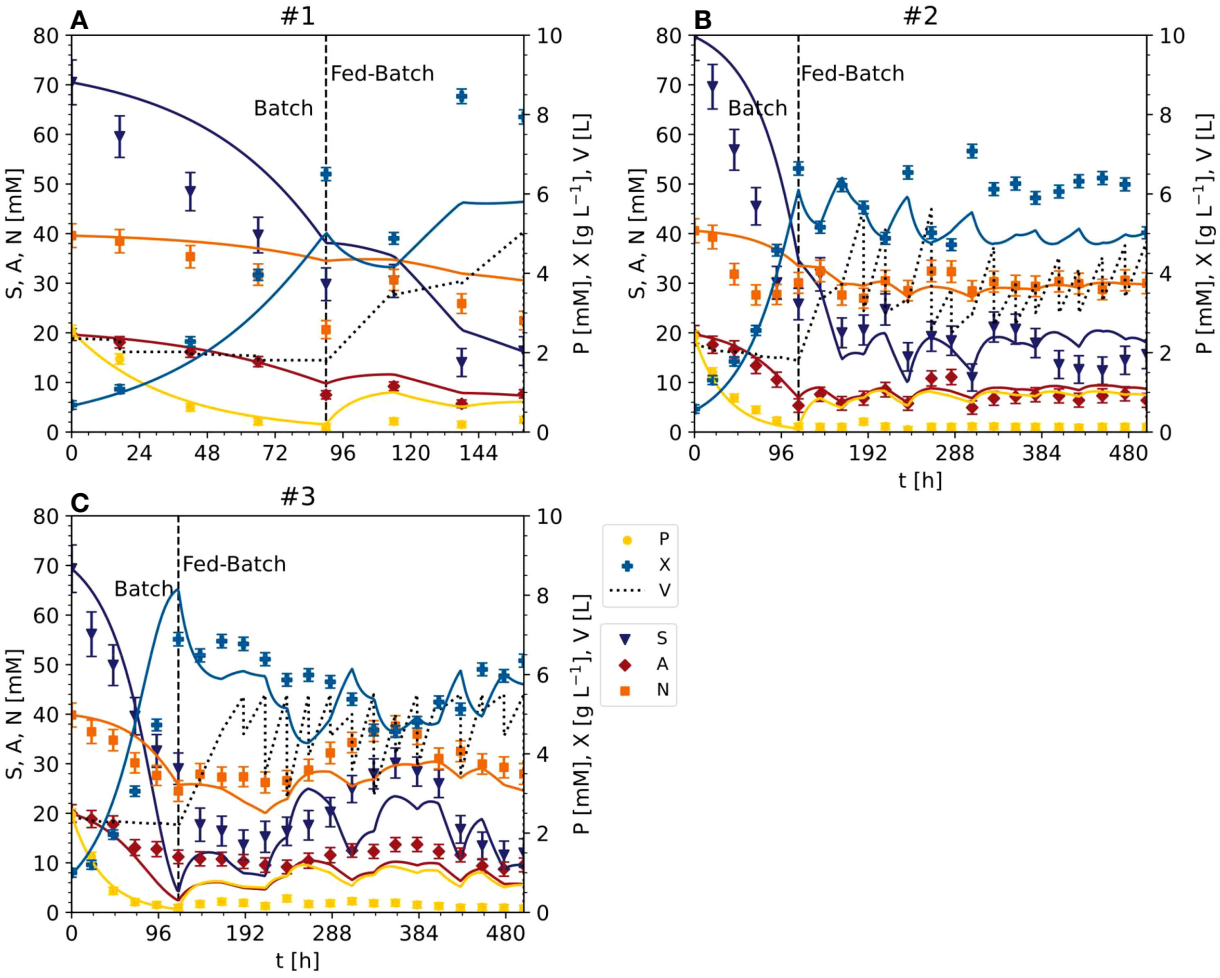
Time trajectories of measured and predicted data (continuous line) for the model set representing the cultivation of BY-2 cells under standard conditions (experiments #1, #2 and #3) **(A–C)** Iteration 1. Measurement uncertainty made of a constant and proportional component (**Table S4**). A, ammonium; F, fructose; G, glucose; N, nitrate; P, phosphate; S, sucrose; V, volume; X, cell dry mass. See **Table S3** for measured initial values.

Parameter identification in equations (9) and (10) for the model (1)-(8) considered all available initial experiments, namely #1, #2 and #3. Therefore, the following quality measures refer to the “training” set, because the same data adopted to establish the model were used to evaluate model performance. For the DM, the MAE was 0.64 [g L^-1^] (**Figure 3**). This corresponds to a nMAE of 0.13. For sucrose, the MAE was 6.58 [mM] (nMAE = 0.22), for ammonium it was 2.21 [mM] (nMAE = 0.22), for nitrate it was 3.48 [mM] (nMAE = 0.11), and for phosphate it was 0.497 [mM] (nMAE = 0.79) (**Figure 3**). Later, the quality measures will be evaluated based on a “test” set, namely on unseen data that has not been used for parameter identification. A higher MAE in the test set than the training set would indicate model overfitting.

Interestingly, in case of all three nutrients (*S, N, A*) the inhibition constants were close the respective saturation constants (*K_r_ ≈ K_Ir_*) (**Table 1**). We had defined the constraint K_r_≥K_Ir_ during parameter identification to reflect a typical activity plateau of enzymes and to exclude an actual reduction in growth at high substrate concentrations. However our observation that parameter optimization favors inhibition constant close to the value of the saturation constants indicates that an actual substrate inhibition, i.e., reduced growth at high concentrations, can occur and therefore allowing for “free-floating” values of K_Ir_ might be a better option in future models.

For sucrose and nitrate, the inhibition and saturation constants were lower than the initial standard concentrations, whereas the constants were higher for ammonium. This suggests that the initial concentrations of sucrose and nitrate can be reduced without significantly changing the specific DM growth (Eq. 4). The predicted phosphate trajectory was satisfactory only during the batch stage (**Figure 3**), i.e., the MAE was ≤0.03 [mM].

### Model-based multi-criteria optimization suggests that reducing the amount of sucrose maximizes the yield and minimizes the process time for most of the Pareto front

Based on the model fitted to the standard BY-2 semi-continuous fermentation setting, we solved a multi-criteria (short time, high yield) optimization problem in which multiple objective functions, were “simultaneously” minimized (Eq. 15):


(15) $$\underset{x\in X}{\min}\;\left(f_{1}\left(x\right),f_{2}\left(x\right),\ldots ,f_{r}\left(x\right)\right)$$

where is the independent variable (e.g., process time, nutrient concentration or starting DM). In the single objective case (i.e., r = 1), this results in one optimal solution. For r > 1, the result is given by the so-called Pareto optimal set in which one objective can only be improved if the values of the other objectives are worsened (Miettinen, 1998). To solve the multi-objective problem (15), it must be transformed into a single objective problem by mathematical scalarization (Finlayson et al., 2015).

**Figure 4:**
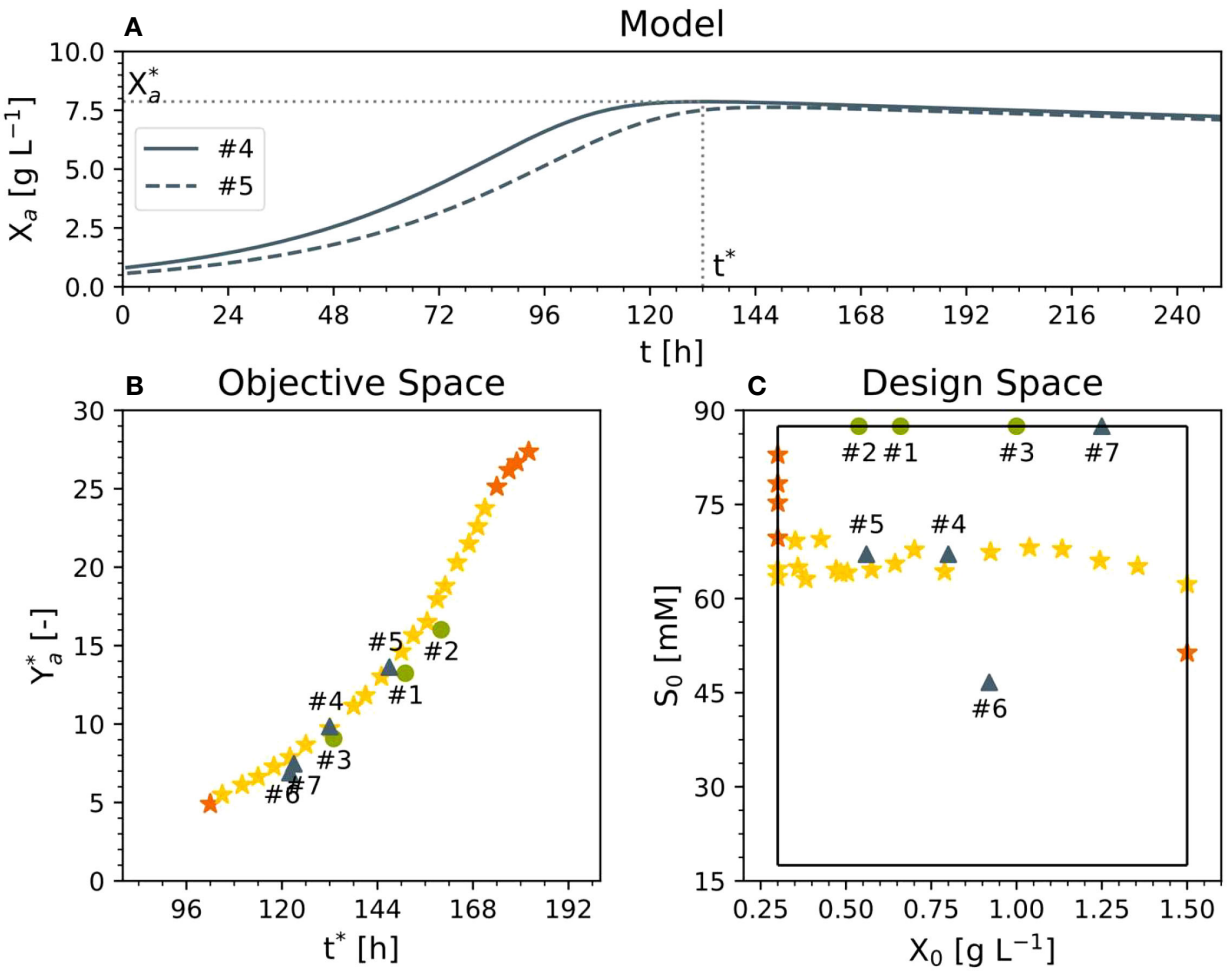
Results of multi-criteria optimization. **(A)** Representative time trajectory of two Pareto solutions relative to active DM. **(B)** Pareto front in the objective and **(C)** design space. Experiments #1–3 were used for model setup, experiments 4 and 5 had an optimal cultivation medium, and experiments 6 and 7 were performed for comparison and model validation purposes (dry mass X, sucrose S, process time t*, active yield Y_a_* at time t*, initial dry mass X_0_, initial sucrose S_0_).


Here, we used two objective functions, i.e., maximized the dimensionless yield of the active DM (Eq. 16), i.e., the gram DM at fermentation end (*X_a_(t)*) divided by the inoculum (i.e., starting) cell DM (*X_0_*):


(16) $$Y_{a}=X_{a}\left(t\right)/X_{0}\overset{t\to min}{\Rightarrow }Y_{a}^{*}=X_{a}^{*}\left(t^{*}\right)/X_{0}$$

evaluated at time t* (i.e., Y_a_^*^), and, simultaneously, minimized the time (t*) at which the maximum active DM was achieved (i.e., the process time), whereby the active DM presented a maximum that was due to both limited cell growth and increasing cell death (**Figure 4A**). To maximize the active DM, we used the sucrose concentration *S_0_* and the inoculum DM concentration (*X_0_*) as variables (i.e., degrees of freedom). The first was varied between 17 and 88 mM while the second was varied between 0.3 and 1.5 g L^-1^ in order to investigate whether lowering the sucrose concentration would be beneficial. The range of the inoculum concentration included the values of the existing experiments. Based on the previous experiments (#1, #2 and #3), we assumed that the initial concentration of the dead DM was zero. The scarcity of data did not allow us to test the model before optimization. Therefore, we limited the degrees of freedom to the inoculum and sucrose, leaving out nitrate and ammonium. Hence, the bi-criteria optimization problem was formulated as follows (Eq. 17):

(17) $$\underset{X_{0},\;S_{0}}{\min}-Y_{a}^{*}\left(X_{0},\;S_{0}\right),\;\;t^{*}\left(X_{0},\;S_{0}\right)$$

The ϵ-constraint or weighted sum methods can be used for scalarization (Finlayson et al., 2015). The weighted sum method identified the convex portion of the Pareto front whereas the ϵ-constraint method identified the remaining (non-convex) section of the front (**Figure 4B**). To ease the solution of the optimization problem, we implemented an iterative approach. First, we identified the maximal Y_a_* (~28) and recorded the associated t*_max_ (the minimal time at which Y_a_* is maximized; ~180 h; top right orange star in **Figure 4B**). Then, we minimized t* under the side condition that the first derivative of Y_a_* is ~0. This corresponded to the minimal time t*_min_ at which Y_a_* peaks, i.e., the earliest maximum of Y_a_* (bottom left orange start in **Figure 4B**; ~96 h). We continued by handling t* as a degree of freedom too (in addition to *X_0_* and *S_0_*). Specifically, in the ϵ-constraint method, the process time t* was allowed to adopt a set of pre-defined values that were uniformly distributed across the range spanned up by t*_min_ and t*_max_ (96–180 h) with a step-width of 3 h (i.e., 28 steps) to cover the entire Pareto front (yellow stars in **Figure 4B**). According to the Pareto front, the shortest t* (i.e., 102 h) is obtained with the highest initial DM concentration (1.5 g L^-1^) (**Figure 4C**). However, the highest DM yield Y_a_* of 27.9 (Eq. 16 with *X_a_* evaluated at t*) would be achieved with the maximum sucrose concentration (88 mM) and the minimum possible initial DM concentration (0.3 g L^-1^) at a time t* of 185 h (**Figure 4C**). This is to be expected because under such conditions the specific concentration of carbohydrate per cell is highest at fermentation start, facilitating the highest relative biomass increase as expressed with Y_a_*. Importantly, alternative definitions of the objective function can be used. For example, the dimensionless biomass yield Y_a_* can be replaced by the biomass concentration at fermentation end *X_a_* (t*).

Moreover, moving along the Pareto front, reducing the sucrose concentration by 25% from 88 mM to 67 mM would increase the active yield and reduce the t* compared to standard conditions (**Figure 4C**). Accordingly, compared to 88 mM sucrose and 0.68, 0.58 and 1.00 g L^-1^ DM in experiments #1, #2 and #3, respectively, we proposed two experiments taken from the Pareto front (#4 and #5) with values of S_0 _= 67 mM and DM = 0.80 and 0.56 g L^-1^, respectively. For comparison and model validation, we also set up two further experiments that were not Pareto optimal (#6 and #7), with 47 mM sucrose/0.92 g L^-1^ DM and 87 mM sucrose/1.25 g L^-1^ DM, respectively, the latter representing the standard BY-2 cell cultivation conditions.

### Reducing the sucrose concentration does not affect biomass formation or volumetric biomass yield when using Pareto optimal conditions

In the optimized setting for the semi-continuous fermentation according to the Pareto front (experiments #4 and #5), the BY-2 cells were cultivated with a starting biomass of 17 g L^-1^ FM (0.80 g L^-1^ DM) and a sucrose concentration of 67 mM (#4), or a starting biomass of 14 g L^-1^ FM (0.56 g L^-1^ DM) and a sucrose concentration of 67 mM (#5), at the beginning of the batch phase (**Figures 2**, **4**; **S3**) in 2-L STRs. We choose a smaller bioreactor volume to accommodate the number of verification runs, i.e., 6 vs the initial 3 runs for model calibration (see also next section). In our hands, both reactor settings (2-L and 5-L) had performed equally in the last 8 years in terms of, for example, oxygen supply, biomass build-up etc. (data not shown).

The validation experiments that were not Pareto optimal started with a biomass of 17 g L^-1^ FM (0.92 g L^-1^ DM) and 47 mM g L^-1^ sucrose (#6) or 22 g L^-1^ FM (1.25 g L^-1^ DM) and 87 mM sucrose (#7), the latter as the standard BY-2 cell cultivation conditions (**Figures 2**, **4** and **S3**). Moreover, because the maximum predicted biomass was not achieved under standard cultivation settings (experiments #1, #2 and #3) because the semi-continuous stage started at 100 g L^-1^ FM, in these experiments the batch stage lasted until the sucrose in the medium was depleted.

In experiments #4 and #5, the FM reached 207.90 g L^-1^ (10.88 g L^-1^ DM) and 181.70 g L^-1^ (10.04 g L^-1^ DM) by the time the sucrose was depleted (within 6 days). The sucrose was hydrolyzed into glucose and fructose, which resulted in a transient peak of 15–17 mM on days 3–4, which declined to <5 mM on day 6. The ammonium was depleted along with the sucrose, but nitrate levels fell by only ~50% from 39 to 19 and 23 mM in experiments #4 and #5, respectively. As observed in experiments #1, #2 and #3, phosphate was almost completely consumed with 2 days during batch fermentation.

In experiment #6, the biomass increased to 172.20 g L^-1^ FM (7.92 g L^-1^ DM) and the starting concentration of sucrose was also depleted in 6 days. Glucose and fructose peaked at 13–16 mM on day 4. Ammonium was depleted by day 6, but nitrate levels fell by only 38%, from 39 to 25 mM.

In experiment #7, a control repeating the standard BY-2 cultivation settings that was not included in the initial model setup, the biomass increased to 270.80 g L^-1^ FM (13.76 g L^-1^ DM) before the sucrose was depleted within 6 days. The transient peak of glucose and fructose (19–22 mM) was observed on day 3 but also declined by day 6. In contrast to sucrose, the ammonium was completely consumed by day 5, and nitrate levels declined by 66% from 39 to 14 mM on day 6. Phosphate was depleted within 3 days.

These data indicate that (1) once the FM exceeded a threshold of 50–60 g L^-1^ FM (3.0–3.5 g L^-1^ DM), the glucose and fructose resulting from sucrose hydrolysis were immediately consumed, (2) the conversion of nitrate to ammonium appeared less efficient than ammonium uptake into cells and its conversion to glutamate and glutamine, and (3) the depletion of sucrose, ammonium and phosphate did not appear to limit the cell growth. To understand the discrepancy between nutrient depletion and cell growth, it may be necessary to include the intracellular metabolism of nutrients in future models. Nevertheless, the relative cell growth was apparently not affected by the lower sucrose concentration and inoculation cell density. Importantly, whereas there was some variability in the fresh-to-dry mass ratio of the inoculum across the set of 9 experiments (21.8 ±4.0, ± standard deviation), that ratio was consistent at the end of the batch phase (19.7 ±1.5, ± standard deviation) indicating that there was no substantial difference in the biomass obtained from the different experiments (i.e., conditions) in terms of water content as it can arise from water uptake into the vacuole.

### Model calibration based on optimized cultivation conditions confirms the biomass yield increase caused by reducing the initial sucrose concentration

**Figure 5:**
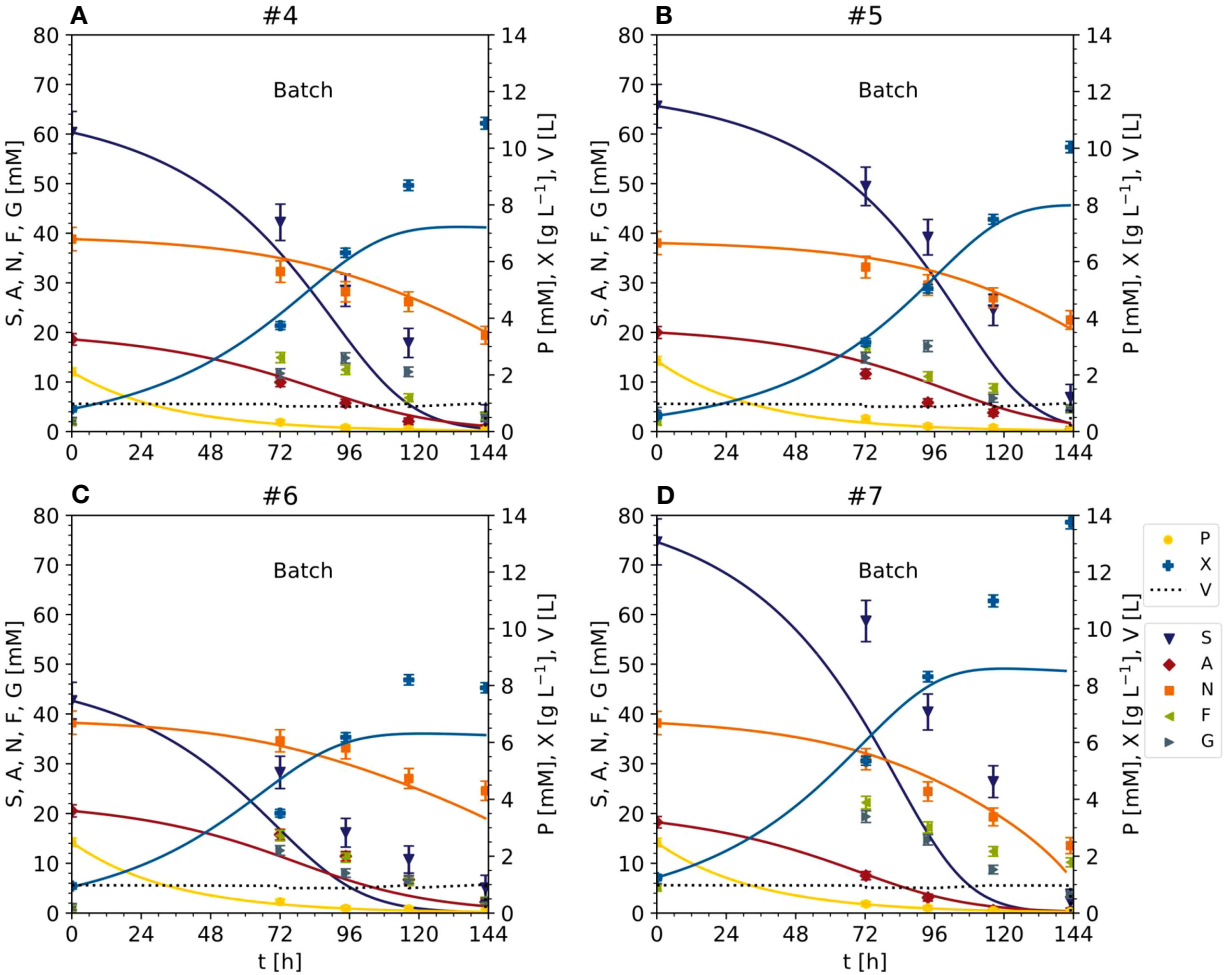
Time trajectories of measured and predicted data (continuous line) for the initial model validation representing the cultivation of BY-2 cells under optimal (experiments #4 and #5) and non-optimal (experiments #6 and #7) conditions **(A–D)** Iteration 2. Measurement uncertainty made of a constant and proportional component (**Table S4**). A, ammonium; F, fructose; G, glucose; N, nitrate; P, phosphate; S, sucrose; V, volume; X, cell dry mass. See **Table S3** for measured initial values.

The data from the Pareto optimal experiments (#4 and #5) and non-optimal comparators (#6 and #7) (**Figures 2** and **S3**) allowed us to test the Monod-type model against unknown data (test set). The MAE was 1.02 [g L^-1^] for DM and 8.18 [mM], 1.38 [mM] and 1.86 [mM] for sucrose, ammonium and nitrate, respectively (**Figures 3**, **5**), confirming in principle that the model could fit the observed nutrient consumption and biomass formation. Therefore, the MAE for nutrients in the test set was lower than or equal to that in the training set, but the MAE for DM was higher in the test set.

**Figure 6:**
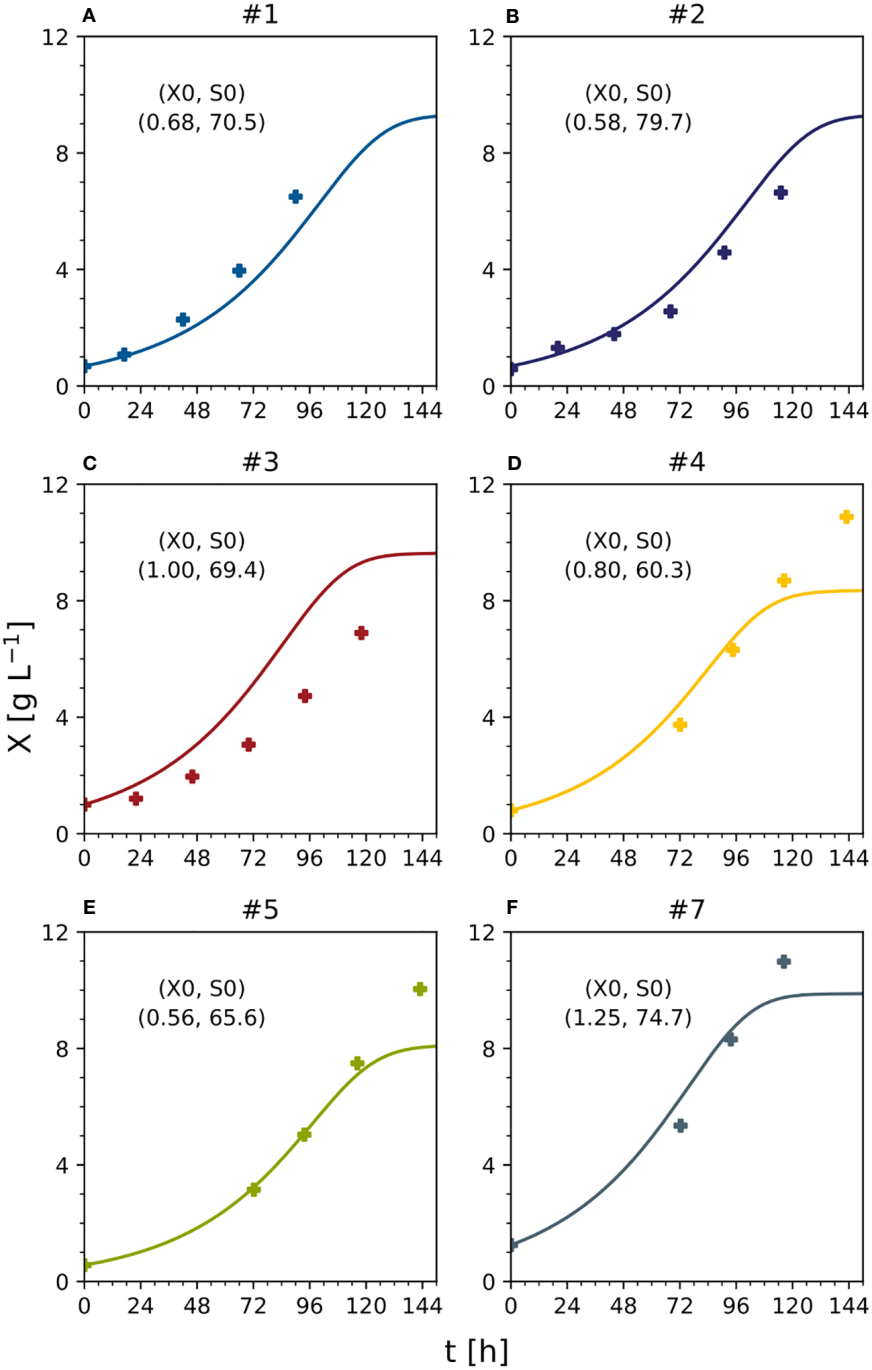
Predicted and measured time trajectories of dry mass under standard, optimal and non-optimal cultivation conditions (batch stage). Text indicates initial DM [g L^-1^] and sucrose [mM] in each experiment. **(A)** Experiment #1. **(B)** experiment #2. **(C)** experiment #3. **(D)** experiment #4. **(E)** experiment #5. **(F)** experiment #7. S_0_ – starting sucrose concentration; t – process time; X – cell dry mass; X_0_ – starting dry mass. See **Table S3** for measured initial conditions of the process parameters.

The model was able to fit the cell growth until the DM reached 8 g L^-1^ but underestimated the real values above this threshold (MAE = 0.30 [g L^-1^] for DM <8 g L^-1^) (**Figure 6**). There are two possible explanations. First, the data used for model fitting (experiments #1, #2 and #3) did not include DM values > 8 g L^-1^. Second, specific cell growth (Eq. 4) was directly dependent on nutrients such as sucrose and ammonium, and the model was not able to calculate DM values when at least one nutrient was close to zero (the case when DM > 8 g L^-1^). Even fitting the model to the data (#4, #5, #6 and #7) did not improve the prediction quality. These considerations, together with the absence of model overfitting (i.e., the MAE of the nutrients in the test set never exceeded that in the training set when DM < 8 g L^-1^), suggested the need for a more complex model, for example, one that includes the hydrolysis of sucrose and/or intracellular metabolism.

**Figure 7:**
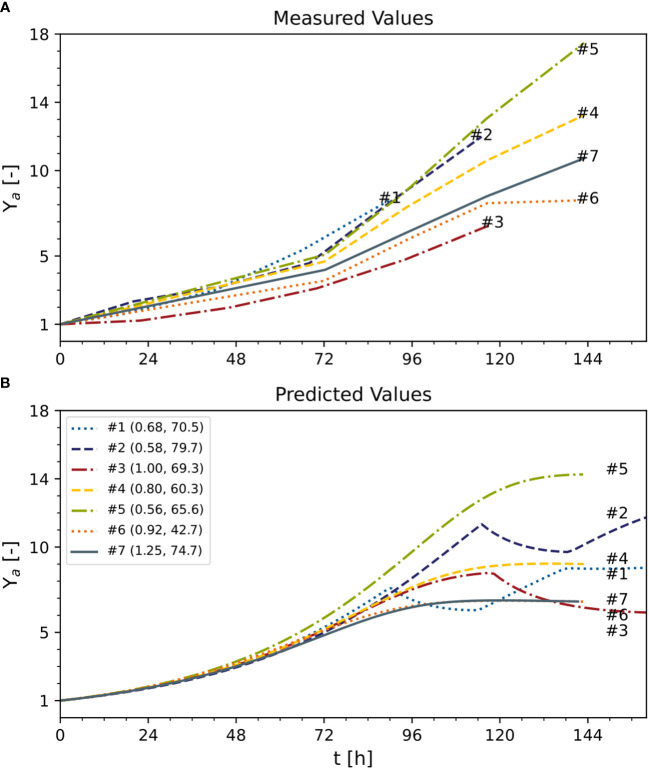
Predicted and measured normalized time trajectories of dry mass under standard optimal and non-optimal cultivation conditions. Text following the experiment number indicates initial DM [g L^-1^] and sucrose [mM] of each experiment. **(A)** Measured values. **(B)** predicted values. Y_a_ – active yield (Eq. 16). Numbers represent the individual experiments. See **Table S3** for measured initial conditions.

To compare DM growth in different experiments with distinct initial inoculum DM values (0.56–1.25 g L^-1^), all measured and predicted DM trajectories were normalized against their initial DM values (**Figure 7**). However, there was a mismatch that caused the model either to underestimate (e.g., experiment #1) or overestimate (e.g., experiment #3) the biomass formation. Due to this mismatch, despite experiments #1, #2, and #3 using the standard initial sucrose concentration (88 mM) and differing only in DM, the model predicted a growing biomass yield for decreasing initial DM values, whereas the measured biomass yields were maximum and minimum at intermediate values of the initial DM (**Figure 7A**). Independently, measured and predicted trajectories confirmed that the model did not fit cell growth accurately when DM >8 g L^-1^ (**Figure 7B**). The assessment of the model’s prediction errors is therefore an important task for future investigations. Nevertheless, we confirmed that a 25% reduction in the concentration of sucrose can increase the volumetric biomass yield by 13%, confirming that model-based optimization of the cultivation medium can improve the performance of BY-2 cell suspension cultures.

### An improved model including glucose and fructose enables the prediction of cell growth at low sucrose concentrations

Given that the model could not predict DM concentrations >8 g L^-1^ when any of the nutrients reached zero, we used a second unstructured model (Eq. 2-3, 5-8, 18-20), accommodating the hydrolysis of sucrose into fructose and glucose (Eq. 18-20). Therefore, the specific growth term depended on glucose and fructose but not on sucrose. Moreover, the influence of ammonium and phosphate were included in the maximum specific growth as additive terms because including them as factors would have zeroed out the growth rate for times >90 h as the phosphate concentration approaches zero, which did not agree with experimental observations as discussed above.


(18) $$\mu =\left(\mu_{m}+\mu_{m}^{A}\frac{A}{A+\delta_{A}}+\mu_{m}^{P}\frac{P}{P+\delta_{P}}\right)\left(\frac{G}{G+K_{G}}\frac{K_{IG}}{G+K_{IG}}+\frac{F}{F+K_{F}}\frac{K_{IF}}{F+KI_{F}}\right)\left(\frac{N}{N+K_{N}}\frac{K_{IN}}{N+K_{IN}}\right)$$

(19) $$V\frac{dF}{dt}+F\frac{dV}{dt}=m_{SF}\alpha \left(\frac{S}{S+K_{S}}\frac{K_{IS}}{S+K_{IS}}\right)X_{a}V-\mu_{F}\left(\frac{F}{F+K_{F}}\frac{K_{IF}}{F+KI_{F}}\right)X_{a}V$$

(20) $$V\frac{dG}{dt}+G\frac{dV}{dt}=m_{SG}\alpha \left(\frac{S}{S+K_{S}}\frac{K_{IS}}{S+K_{IS}}\right)X_{a}V-\mu_{G}\left(\frac{G}{G+K_{G}}\frac{K_{IG}}{G+K_{IG}}\right)X_{a}V$$

The sucrose to glucose or fructose DM conversion parameters (m_SG_ and m_SF_) were set at 0.526 because one mole of sucrose always produces one mole each of glucose and fructose (Puad et al., 2017). This model is defined hereafter as the “improved model”. It has 20 parameters to identify, including the death rate constant $k_{d}$ , maximum specific growth rate *μ_m_*, maximum specific growth rate for ammonium and phosphate *μ^A^_m,_ μ^P^_m,_* saturation and inhibition constants *K_h_* and *K_Ih_* , hydrolysis constant α, and consumption rates *μ_h_*. Coefficients *δ_A_, δ_P_* can be interpreted as the minimal ammonium and phosphate concentration in the medium at which cell growth occurs. They were assumed known in the context of this study to limit the model complexity and avoid overfitting and their values were set to 10% of the starting concentrations. This allowed a nearly constant growth contribution when they are not depleted as observed in the experiments. As the number of experiments available for model calibration iteratively increase, the two parameters can be fitted too.

We used K-fold cross-validation to show the better prediction capability of the improved model compared to the initial one (Stone, 1974). With *K* = 4 full data sets available, i.e., all experiments performed so far in which fructose and glucose were measured: #4-7. Experiments #1-3 were not considered since the improved model would need the initial concentrations of fructose and glucose. The *k-th* fold consists of splitting the data so that the *k-th* set is used for testing, while the remaining *K*–1 are used for model setup (i.e., model training). It follows that for each model (i.e., initial and improved), *k* parameter identifications were performed (each relying on 3 experiments) with the performance measures estimated on the corresponding test set. The improved model was supposed to have a lower average validation (i.e., test) error than the initial one:


(21) $$aMAE_{i}=\frac{1}{K}\sum_{k=1}^{K}=1K\;MAE_{i}^{k}\left(\theta_{k}^{*}\right)$$

Analogously:


(22) $$anMAE_{i}=aMAE_{i}/{\bar{y}}_{i}$$

Whereas the MAE is a quality measure that refers to a single set of model parameters θ*, the aMAE is the average MAE over different models (i.e., different parameter sets). The following analysis allowed a fair comparison between models and the aMAE provided an estimation of the modeling prediction error on unseen data.

**Figure 8:**
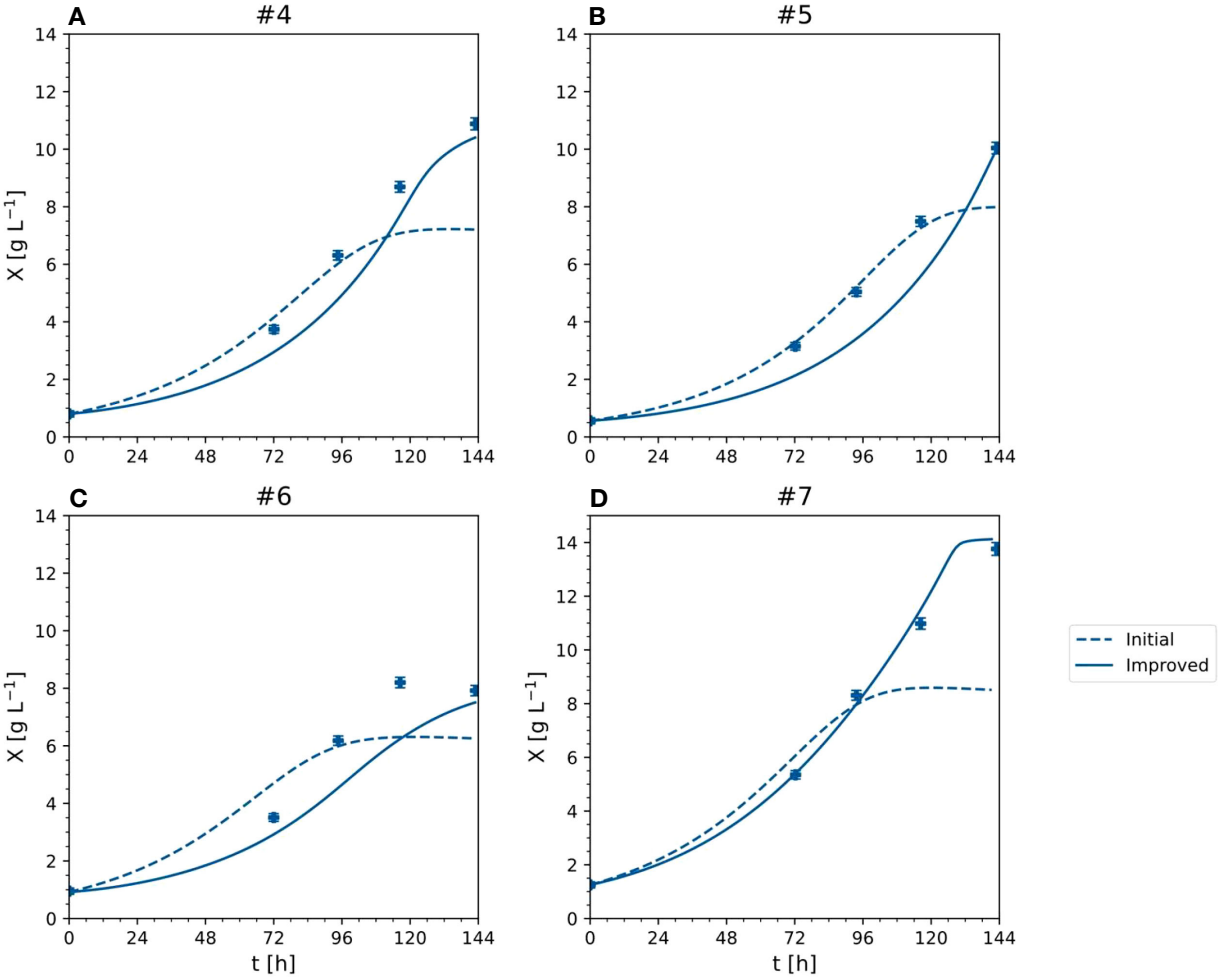
X - cell dry mass time trajectories of measured and predicted data for the initial and improved models representing the cultivation of BY-2 cells under optimal and non-optimal conditions (experiment #4, … #7). **(A–D)** Iteration 2.

Compared to the initial model, the improved model had a lower or equal aMAE_i_ for all outputs (**Table 2**). The most significant improvements occurred for the DM (the anMAE_X_ dropped from 0.214 to 0.149) and sucrose (the aMAE_S_ was 30% lower in the improved model). The average error of the prediction for ammonium and nitrate remained the same. Such results indicated the better modeling capability of the improved model, in particular the ability to capture high DM values (**Figure 8**). For DM greater than 8 [g L^-1^], anMAE_X_ dropped from 0.371 to 0.097 (**Table 2**).

**Table 2:** Average and average normalized test MAE values for initial and improved models.

Model	aMAE_X_ [g L^-1^] (anMAE_X_ [-])	aMAE_X>8_ [g L^-1^] (anMAE_X>8_ [-])	aMAE_S_ [mM] (anMAE_S_ [-])	aMAE_A_ [mM] (anMAE_A_ [-])	aMAE_N_ [mM] (anMAE_N_ [-])
Initial	1.069 (0.214)	1.850 (0.371)	5.841 (0.198)	1.983 (0.194)	3.082 (0.101)
Improved	0.741 (0.149)	0.488 (0.097)	4.541 (0.153)	1.808 (0.177)	3.232 (0.106)

**Figure 9:**
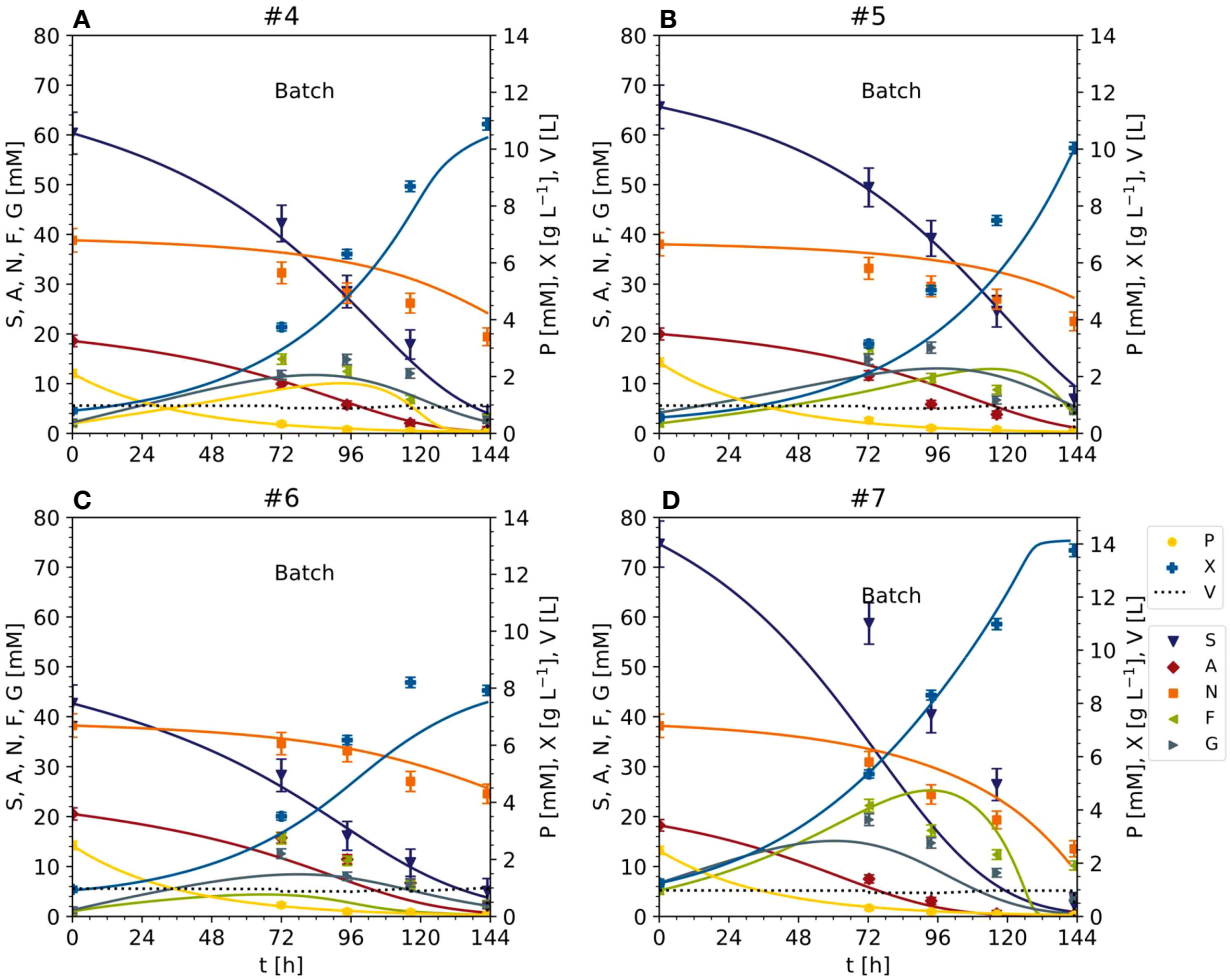
Time trajectories of measured and predicted data (continuous line) for the improved model representing the cultivation of BY-2 cells under optimal and non-optimal conditions (experiment #4, … #7). **(A–D)** Iteration 2. Measurement uncertainty made of a constant and proportional component (**Table S4**). A, ammonium; F, fructose; G, glucose; N, nitrate; P, phosphate; S, sucrose; V, volume; X, cell dry mass. See **Table S3** for measured initial values.

**Figure 10:**
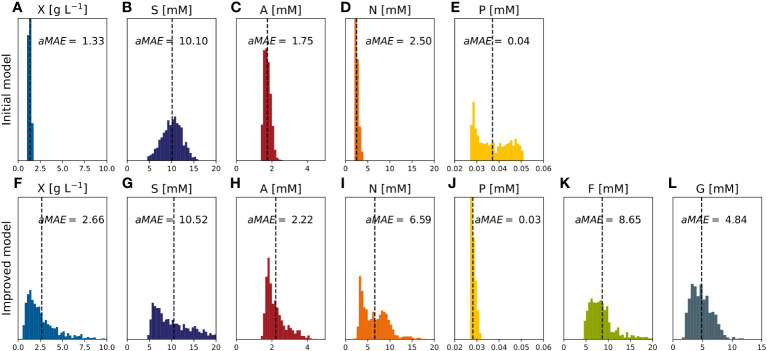
MAE of model factors describing the model-data mismatch obtained with random parameter set values (~1000) uniformly sampled within the confidence intervals (experiments #4,…,#7). **(A–E)** Initial model. **(F–L)** Improved model. A, ammonium; F, fructose; G, glucose; N, nitrate; P, phosphate; S, sucrose; X, cell dry mass.

The improved model was then retrained with all available full data sets (i.e., experiments #4-7) resulting in the proposed identified parameters (**Table 3**). The MAE_i_ estimated on the training data (**Figure 9**) was 0.58 [g L^-1^] for DM and 4.31, 1.35, 3.03 and 0.02 [mM] for sucrose, ammonium, nitrate, and phosphate respectively. The MAE was equal or lower (**Figure 9**) compared to the initial model (**Figure 3**) in all model outputs. Nevertheless, this did not necessarily imply a better performance because different data sets were used and the MAE relied on training data. The MAEs for fructose and glucose, not included in the initial model, were 3.81 and 2.29 [mM] with nMAE values of 0.409 and 0.261, respectively. Among the nutrients, fructose showed the highest nMAE and its concentration was thus most difficult to predict (**Figure 9**). The reason for the low predictability is unknown but may reflect a staged metabolism that is not solely dependent on fructose alone and would require a modification of the kinetic model (Eq. 5). CIs were in the same order of magnitude as the values of the identified parameters (**Table 3**). Such a result is not desirable because it indicates a moderate predictive power of the underlying model and can hamper the identification of relevant differences between samples/conditions (O’Brien and Yi, 2016). However, wide CIs were expected because of the large number of parameters and the small data set. The effect of the wide CIs on the predictive ability of the models was assessed by generating 1,000 random sets of model parameter values θ, uniformly sampled from inside the CIs. These sets were then used to create model predictions and estimate the model-data mismatch based on MAE (Eq. 12), aMAE (Eq. 21) and anMAE (Eq. 22) for each model factor using experiments #4-7 as references (**Figure 10**). In case of the initial model, the anMAE was equal to 26%, 17%, 8%, 5% for DM, ammonium, nitrate, and phosphate, respectively, which was less than the sum of the model independent process parameter uncertainties (**Table S4**), which was approximately 27%. For this sum, constant and proportional measurement uncertainties of each process parameter were unified under a single relative uncertainty, namely 3% for DM, 6% for each nutrient. This observation suggested that the model still retained predictive validity when model parameters *θ* were inside the CIs because the variability of the model predictions were in the same range as the uncertainty of the independent process variables. The sole exception in this context was sucrose, which had an anMAE of 34%, which was higher than the sum of process parameter uncertainties.

**Table 3:** Parameter ranges and identified optimal parameter values for the improved model (experiments #1 to #7 were used for model setup).

Parameter	Description	Unit	Min	Max	Optimal	90% CI
$k_{d}$	death rate constant	h^-1^	0	0.01	5.44 × 10^-4^	6.2 × 10^-5^
$\mu_{m}$	maximum specific growth rate	h^-1^	0	10	0.162	0.17
$\mu_{m}^{A}$	maximum specific growth rate ammonium	h^-1^	0	10	0.578	0.48
$\mu_{m}^{P}$	maximum specific growth rate phosphate	h^-1^	0	10	2.1 × 10^-5^	0.37
$K_{S}$	Sucrose saturation constant	g L^-1^	0	100	72.53	73.4
$K_{IS}$	Sucrose inhibition constant	g L^-1^	0	100	100.0	73.3
$K_{A}$	Ammonium saturation constant	g L^-1^	0	1	0.221	0.22
$K_{IA}$	Ammonium inhibition constant	g L^-1^	0	1	0.221	0.065
$K_{N}$	Nitrate saturation constant	g L^-1^	0	5	0.835	0.25
$K_{IN}$	Nitrate inhibition constant	g L^-1^	0	5	1.0	0.06
$K_{F}$	Fructose saturation constant	g L^-1^	0	30	1.108	0.71
$K_{IF}$	Fructose inhibition constant	g L^-1^	0	30	1.404	0.51
..	Glucose saturation constant	g L^-1^	0	30	18.0	17.1
$K_{IG}$	Glucose inhibition constant	g L^-1^	0	30	18.0	12.0
α	Sucrose hydrolysis constant	h^-1^	0	1	0.396	0.24
$\mu_{A}$	Ammonium consumption rate	h^-1^	0	1	6.9 × 10^-3^	1.9 × 10^-3^
$\mu_{N}$	Nitrate consumption rate	h^-1^	0	1	1.71 × 10^-2^	2.7 × 10^-2^
$\mu_{F}$	Fructose consumption rate	h^-1^	0	1	8.34 × 10^-2^	2.9 × 10^-2^
$\mu_{G}$	Glucose consumption rate	h^-1^	0	1	0.273	0.26
$\mu_{P}$	Phosphate consumption rate	h^-1^	0	1	2.68 × 10^-2^	3.2 × 10^-3^

**Figure 11:**
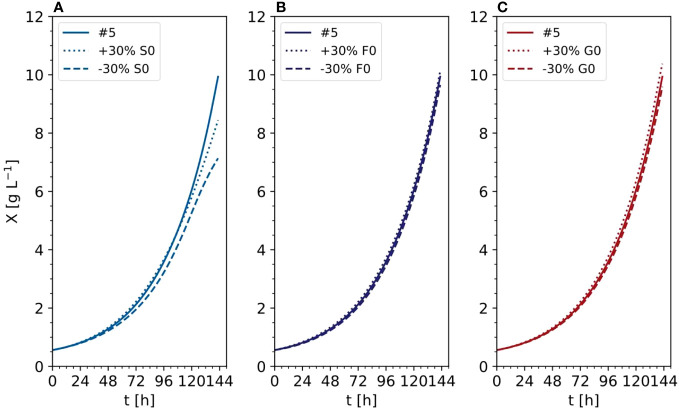
Time trajectories of predicted data with the improved model comparing the influence of initial carbon sources **(A)** Sucrose, **(B)** Fructose, **(C)** Glucose) in the DM (experiment #5). X, cell dry mass.

In the improved model, the anMAE values were 53%, 35%, 21%, 21%, 4%, 42%, 24%, for DM, sucrose, ammonium, nitrate, phosphate, fructose, and glucose, respectively (**Figure 10**), whereas the sum of the modeled independent process parameter uncertainties was 39%. Like the initial model, the improved model suggested that, with respect to the standard cultivation conditions, a 30% reduction of sucrose in the medium is beneficial for the DM growth (**Figure 11A**, e.g., experiment #5). Also, glucose seemed to be preferred over fructose as a carbon source (**Figures 11B, C**) which was qualitatively confirmed in experiments #8 and #9. Therefore, including the individual carbon sources in the model allowed an improvement of the prediction compared to the model accounting only for the “precursor” sucrose, that is not taken up by the cells directly, but only after breakdown to the monosaccharides.

**Figure 12:**
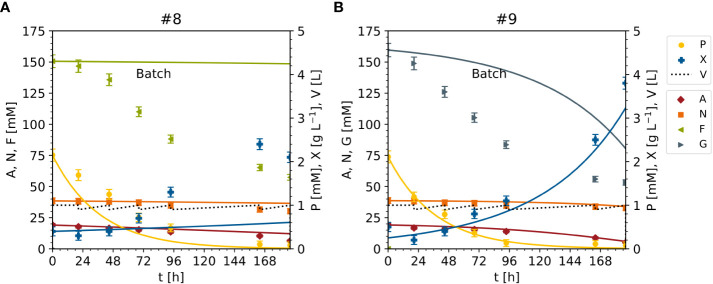
Time trajectories of measured and predicted data (continuous line) for the model set up representing the cultivation of BY-2 cells under non-optimal culture conditions with either fructose (experiment #8) or glucose (experiment #9) as the sole carbon source for improved model validation. **(A, B)** Iteration 2. A – ammonium. F – fructose. G – glucose. N – nitrate. P – phosphate. S – sucrose. V – volume. X – cell dry mass.See **Table S3** for measured initial values. Measurement uncertainty made of a constant and proportional component (**Table S4**).

Finally, the model was adopted to predict DM and nutrients for experiments #8 and #9 (unseen data), in which sucrose was replaced with either fructose (#8) or glucose (#9) as the sole carbon source (**Figures 2**, **12**, **S4**). The DM prediction was mediocre (MAE_X_ = 0.37 [g L^-1^]), and were very good for ammonium (MAE_A_ = 0.88 [mM]) and nitrate (MAE_N_ = 0.92[mM]), but poor for fructose or glucose (MAE_F_ = 25.06[mM], MAE_G_ = 19.60 [mM]) (**Figure 12**). Indeed, MAE_F_ and MAE_G_ were one order of magnitude larger than in the model setup (**Figure 12**). The decline in the concentrations of fructose and glucose was underestimated by the model (50 mM measured compared to 100 mM predicted), possibly because the model was fitted against data sets in which the fructose and glucose concentrations were 0–25 mM (**Figure S3**), whereas in experiments #8 and #9 the concentrations were 150 mM (**Figure S4**). Nevertheless, the inclusion of fructose and glucose in the model enabled the prediction of cell growth and biomass formation at high cell densities and low sucrose concentrations, and improved the modeling capability with respect to the initial model (**Table 3**). To increase the model accuracy further, the unstructured model might be converted to a structured one that includes the intracellular metabolism of nutrients.

## Conclusions

We have shown that adopting the iterative “experiment-modeling-optimization” workflow achieved a 13% increase in the growth of tobacco BY-2 cell suspension cultures while reducing the sucrose concentration in the cultivation medium by 25%. This was based on a mechanistic unstructured segregated Monod-type model using the sucrose concentration as a relevant parameter. However, the initial model could not predict cell growth at high cell densities, which was resolved by an improved model that included glucose and fructose as sucrose hydrolysis products. Moreover, cell growth could only be fitted if the nutrients did not reach zero, so we removed phosphate from the model because this was completely depleted. This shortcoming might be addressed by a structured model that includes intracellular nutrient metabolism. Nevertheless, the model suggested that sucrose concentration and inoculation cell density can be reduced to maximize the yield, which we confirmed in validation experiments. Mechanistic models can therefore be used to maximize the productivity of cell suspension cultures while reducing upstream production costs. This may be applicable not only to plant cells, but also to microbial and mammalian cell cultures used for the production of biopharmaceuticals.

## Data availability statement

The data supporting the findings of this study are available from the corresponding author upon reasonable request.

## Author contributions

HN and JB designed the experiments. HN and JL conducted the experiments and pre-processed the data. MaB, KT and MiB analyzed the data and built the models. HN and MB wrote the manuscript. CC, MiB, and JB revised the manuscript and secured the funding. All authors contributed to the article and approved the submitted version.

## Footnotes

1 https://julialang.org/

2 https://coin-or.github.io/Ipopt/OPTIONS.html
