# Supplementary material for: Simulation and optimization of nutrient uptake and biomass formation using a multi-parameter Monod-type model of tobacco BY-2 cell suspension cultures in a stirred-tank bioreactor
Source: Front Plant Sci. 2023 Oct 31;14:1183254. doi: 10.3389/fpls.2023.1183254 (PMC10731461; doi:10.3389/fpls.2023.1183254)
Supplement: Supplementary file 1 [file DataSheet_1.docx]

**Supplementary Material**


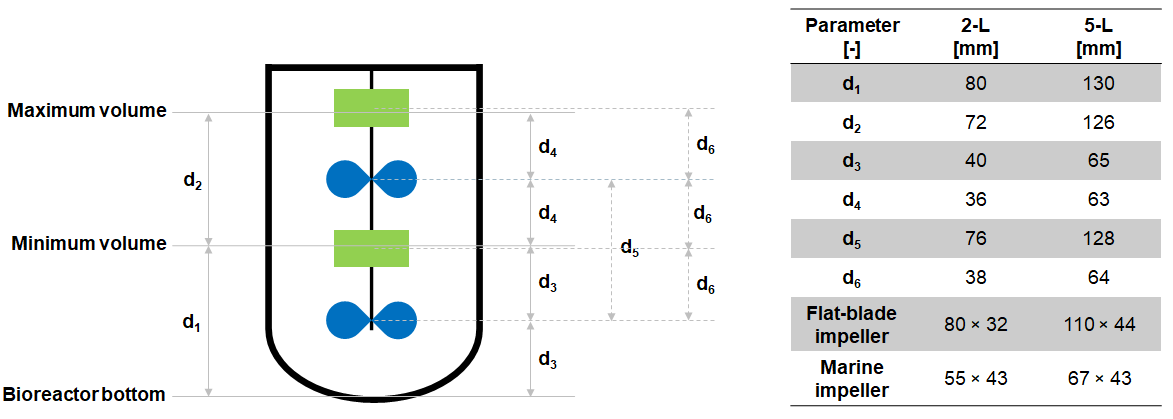


**Figure S1.** Stirrer configuration and positioning in 2-L and 5-L bioreactors used for BY-2 cell cultivation. Flat-blade impellers are shown in green, marine impellers are colored in blue. The minimal operation volumes were 1.0 L and 2.5 L whereas maximal volumes were 2.0 and 5.0 L for the 2-L and 5-L reactors respectively.


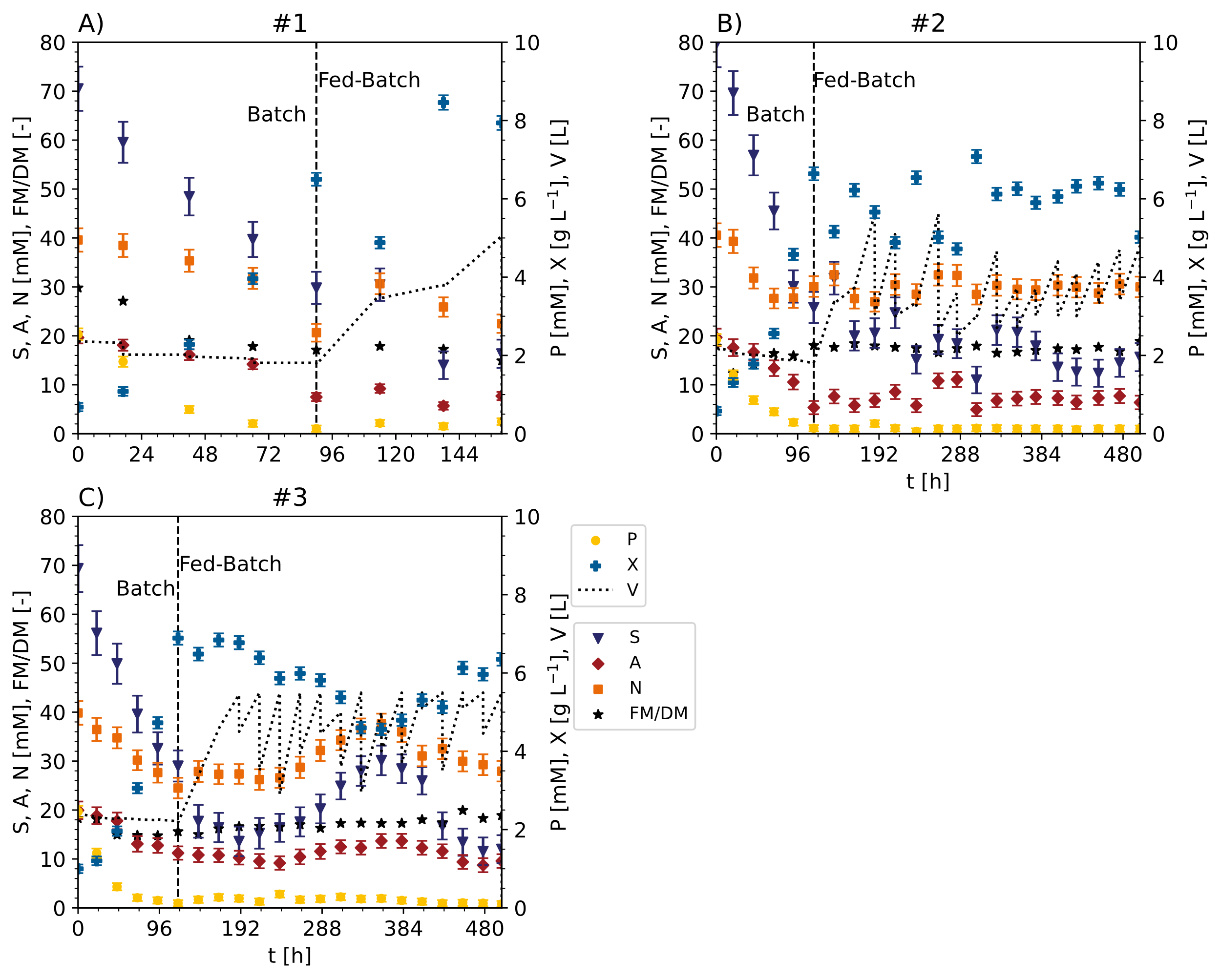


**Figure S2.** Nutrient consumption and cell growth/biomass formation in the individual experiments under standard cultivation conditions for model setup (experiments #1 to #3). **(A–C)** Iteration 1. See Table S4 for measurement uncertainty. A – ammonium. F – fructose. G – glucose. N – nitrate. P – phosphate. S – sucrose. V – volume, X – cell dry mass, FM/DM – fresh-dry mass ratio. See Table S3 for measured initial values.


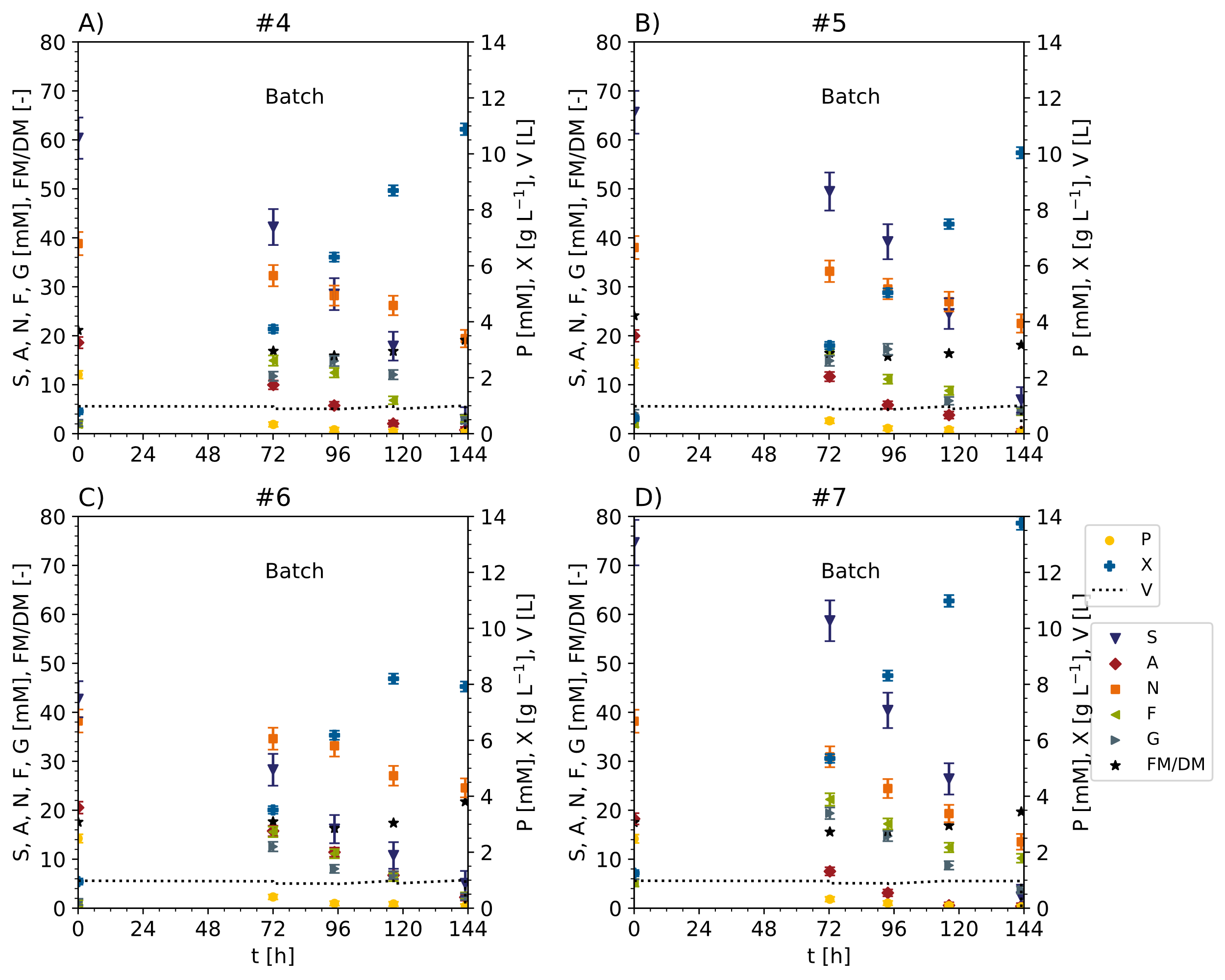


**Figure S3.** Nutrient consumption and cell growth/biomass formation in the individual experiments with optimal (experiments #4 and #5) and non-optimal (experiments #6 and #7) culture medium for model validation. **(A–D)** Iteration 2. See Table S4 for measurement uncertainty. A – ammonium. F – fructose. G –glucose. N – nitrate. P – phosphate. S – sucrose. V – volume. X – cell dry mass, FM/DM – fresh-dry mass ratio. See Table S3 for measured initial values.


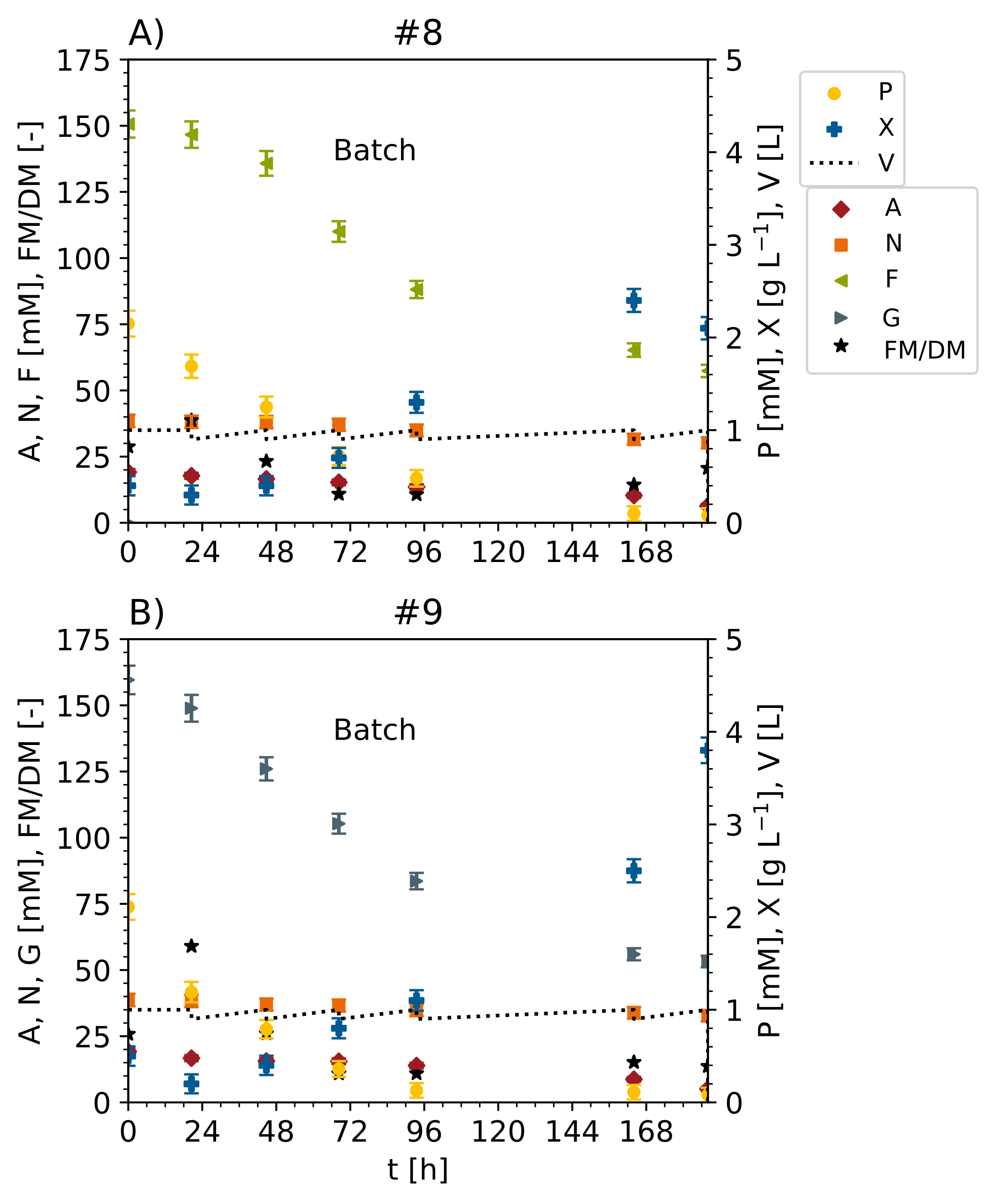


**Figure S4.** Nutrient consumption and cell growth/biomass formation in the individual experiments with non-optimal culture conditions and either fructose (experiment #8) or glucose (experiment #9) as the sole carbon source for validation of the improved model. **(A,B)** Iteration 2. See Table S4 for measurement uncertainty. A – ammonium. F – fructose. G – glucose. N – nitrate. P – phosphate. S – sucrose. V – volume. X – cell dry mass, FM/DM – fresh-dry mass ratio. See Table S3 for measured initial values.

**Table S1.** Iterative workflow for model-based bioprocess engineering. In the first iteration, initial experiments were carried out to collect starting data (e.g., for nutrient consumption, cell growth and biomass formation), a model was set up in order to identify relevant parameters (e.g., nutrients), and the model, once embedded in an optimization frame, was used to predict optimal process conditions (e.g., initial nutrient concentrations). In the second iteration, these optimized process conditions were experimentally verified and used to update the model calibration and/or process and for model improvement.

| **Workflow** | **Iteration 1** | **Iteration 2** |
| --- | --- | --- |
| Experiment | Initial process for data collection | Optimized process for model validation |
| Modeling | Model set-up and parameter identification | Model improvement to increase prediction accuracy |
| Optimization | Model-based process optimization | Model-based process optimization |

**Table S2.** Setup of the 2-L and 5-L STRs used in this study.

| **Parameter** | **Unit** | **2-L** | **5-L** |
| --- | --- | --- | --- |
| Temperature | °C | 26 | 26 |
| Aeration rate | L min^-1^ / vvm | 0.5 / 0.2 | 1.0 / 0.2 |
| Dissolved oxygen (dO_2_) | % | 20 | 20 |
| Stirred speed min | rpm | 160 | 100 |
| Stirred speed max | rpm | 320 | 200 |
| Culture volume min | L | 1.0 | 2.5 |
| Culture volume max | L | 2.0 | 5.0 |
| Antifoam (vegan Struktol SB2239A)^a^ | % v v^-1^ | 0.01 | 0.01 |
| Inoculation cell density | g FM L^-1^ | 20 | 20 |
| Experiments | # | 4–9 | 1–3 |

FM – fresh mass; rpm – revolutions per minute; vvm – volume per volume and minute; ^a^ from Schill + Seilacher “Struktol” GmbH, Hamburg, Germany.

**Table S3.** Measured initial values of cell dry mass and nutrients in different experiments as used for model calibration. The intended set points are given in the text of the methods section.

| **Experiment** | **X_0_**  **[g L^-1^]** | **S_0_**  **[mM]** | **F_0_**  **[mM]** | **G_0_**  **[mM]** | **A_0_**  **[mM]** | **N_0_**  **[mM]** | **P_0_**  **[mM]** |
| --- | --- | --- | --- | --- | --- | --- | --- |
| #1 | 0.68 | 70.49 | n/a | n/a | 19.64 | 39.59 | 2.54 |
| #2 | 0.58 | 79.69 | n/a | n/a | 19.68 | 40.58 | 2.40 |
| #3 | 1.00 | 69.35 | n/a | n/a | 19.95 | 39.84 | 2.46 |
| #4 | 0.80 | 60.35 | 1.93 | 2.19 | 18.59 | 38.82 | 2.11 |
| #5 | 0.56 | 65.64 | 1.89 | 4.17 | 19.98 | 38.02 | 2.50 |
| #6 | 0.92 | 42.67 | 1.00 | 1.28 | 20.53 | 38.23 | 2.49 |
| #7 | 1.25 | 74.65 | 5.17 | 6.70 | 18.25 | 38.18 | 2.48 |
| #8 | 0.40 | n/a | 150.64 | n/a | 19.11 | 38.51 | 2.15 |
| #9 | 0.50 | n/a | n/a | 159.59 | 19.18 | 38.69 | 2.11 |

A – ammonium, F – fructose, G – glucose, N – nitrate, P – phosphate, S – sucrose, X – cell dry mass, n/a – not analyzed.

**Table S4.** Constant (u^0^) and proportional (u^%^) measurement uncertainty for the process parameters monitored during BY-2 cell fermentation.

| **Uncertainty component** | **X** | **S** | **F** | **G** | **A** | **N** | **P** |
| --- | --- | --- | --- | --- | --- | --- | --- |
| u^0^ | 0.05 [g L^-1^] | 2.5 [mM] | 0.6 [mM] | 0.6 [mM] | 0.6 [mM] | 1.2 [mM] | 0.08 [mM] |
| u^%^ [-] | 2 | 3 | 3 | 3 | 3 | 3 | 3 |

A – ammonium, F – fructose, G – glucose, N – nitrate, P – phosphate, S – sucrose, X – cell dry mass.
